# *Porphyromonas gingivalis* Uses Specific Domain Rearrangements and Allelic Exchange to Generate Diversity in Surface Virulence Factors

**DOI:** 10.3389/fmicb.2017.00048

**Published:** 2017-01-26

**Authors:** Stuart G. Dashper, Helen L. Mitchell, Christine A. Seers, Simon L. Gladman, Torsten Seemann, Dieter M. Bulach, P. Scott Chandry, Keith J. Cross, Steven M. Cleal, Eric C. Reynolds

**Affiliations:** ^1^Oral Health Cooperative Research Centre, Melbourne Dental School, Bio21 Institute, University of MelbourneVIC, Australia; ^2^Victorian Life Sciences Computation InitiativeCarlton, VIC, Australia; ^3^CSIRO Food and NutritionWerribee, VIC, Australia

**Keywords:** periodontal pathogen, *Porphyromonas gingivalis*, genetic diversity, specific domain rearrangement, surface virulence factors

## Abstract

*Porphyromonas gingivalis* is a keystone pathogen of chronic periodontitis. The virulence of *P. gingivalis* is reported to be strain related and there are currently a number of strain typing schemes based on variation in capsular polysaccharide, the major and minor fimbriae and adhesin domains of Lys-gingipain (Kgp), amongst other surface proteins. *P. gingivalis* can exchange chromosomal DNA between strains by natural competence and conjugation. The aim of this study was to determine the genetic variability of *P. gingivalis* strains sourced from international locations over a 25-year period and to determine if variability in surface virulence factors has a phylogenetic basis. Whole genome sequencing was performed on 13 strains and comparison made to 10 previously sequenced strains. A single nucleotide polymorphism-based phylogenetic analysis demonstrated a shallow tri-lobed phylogeny. There was a high level of reticulation in the phylogenetic network, demonstrating extensive horizontal gene transfer between the strains. Two highly conserved variants of the catalytic domain of the major virulence factor the Kgp proteinase (Kgp_cat_I and Kgp_cat_II) were found. There were three variants of the fourth Kgp C-terminal cleaved adhesin domain. Specific variants of the cell surface proteins FimA, FimCDE, MfaI, RagAB, Tpr, and PrtT were also identified. The occurrence of all these variants in the *P. gingivalis* strains formed a mosaic that was not related to the SNP-based phylogeny. In conclusion *P. gingivalis* uses domain rearrangements and genetic exchange to generate diversity in specific surface virulence factors.

## Introduction

Chronic periodontitis is an inflammatory disease associated with bacteria which results in destruction of the tooth's supporting tissues including the alveolar bone (Wiebe and Putnins, 2000). The disease is associated with specific bacteria in a polymicrobial biofilm, subgingival plaque, accreted to the surface of the root of the tooth. In the USA, 38% of the adult population 30 years and older and 65% of adults 65 years and older have either severe or moderate periodontitis (Eke et al., 2012). Epidemiological surveys have shown that clinical indicators of periodontitis are linked to an increased risk of cardiovascular diseases, certain cancers (orogastrointestinal tract and pancreas), rheumatoid arthritis, adverse pregnancy outcomes, and other systemic diseases related to the regular bacteraemia and chronic inflammation associated with the disease (Genco and Van Dyke, 2010; Lundberg et al., 2010; Linden et al., 2013; Madianos et al., 2013; Tonetti and Van Dyke, 2013).

*P. gingivalis* is a keystone pathogen of chronic periodontitis that dysregulates the host immune response to favor the proliferation of the polymicrobial biofilm thereby disrupting homeostasis with the host to cause dysbiosis and disease (Hajishengallis et al., 2012). The closely related *Porphyromonas gulae* is proposed to play a similar role to *P. gingivalis* in the development of periodontitis in dogs (Fournier et al., 2001; Lenzo et al., 2016).

The pathogenicity of *P. gingivalis* is attributed to a number of surface-associated virulence factors that include cysteine proteinases (gingipains), fimbriae, haem-binding proteins, and outer membrane transport proteins amongst others. In particular, the cysteine endoproteinases, the Arg-specific gingipains (RgpA and RgpB) and Lys-specific gingipain (Kgp) have multiple effects on both the innate and adaptive immune responses (Popadiak et al., 2007; Bostanci and Belibasakis, 2012). All three gingipain polyproteins contain a signal peptide of ~22 amino acids in length, an unusually long propeptide of over 200 amino acids in length, and a catalytic domain of ~360 amino acids. In addition RgpA and Kgp have repetitive haemagglutinin-adhesin (HA) domains in the polypeptide region C-terminal to the catalytic domains. Some of these have been alternatively described as C-terminal adhesin domains or cleaved adhesin domains (CADs) and some are DUF 2436 domains (O'Brien-Simpson et al., 2003; Li et al., 2010). The RgpA and Kgp precursor proteins are cleaved into multiple domains that remain non-covalently associated forming large outer membrane protein complexes (Bhogal et al., 1997). The Kgp_cat_ domain structure consists of a central 10 stranded β-sheet surrounded by 10 α-helices forming an α*-*β sandwich domain. An immunoglobulin superfamily-like domain comprising six antiparallel β-strands, is situated on the opposite side from the substrate binding face of the α-β domain (de Diego et al., 2014; Gorman et al., 2015). RgpB lacks the adhesin domains and is located in a monomeric form on the outer membrane and has a structure similar to Kgp_cat_ (Eichinger et al., 1999). *P. gingivalis* strains also contain the large polyprotein HagA which has multiple repeating CADs that associate with other cell surface proteins including the gingipains (Kozarov et al., 1998; Frazer et al., 2006).

Fimbriae facilitate adherence to both host cells and other bacterial members of the plaque microbiota. *P. gingivalis* has at least two types of fimbriae, including the major fimbriae encoded by *fimA-E* and the minor fimbriae encoded by *mfa1-4*. The major fimbriae are involved in colonization and cellular invasion and both types of fimbriae have proinflammatory capacity (Yilmaz et al., 2002; Bostanci and Belibasakis, 2012). The major fimbriae in *P. gingivalis* are comprised of the FimA fimbrillin structural protein and FimCDE accessory structural proteins that are involved in adhesion specificity. There are six reported FimA types, types I, Ib, II-V based on identified genome sequences with predicted molecular masses ranging from 41 to 49 kDa (Amano et al., 1999, 2004). The *P. gingivalis* minor fimbriae are encoded by *mfa1-4* and two *mfa1* genotypes encoding two distinct Mfa1 fibrillin monomers have been described (Nagano et al., 2015).

Virulence factors such as the fimbriae and gingipains are the basis for typing methods used to identify disease-associated strains (Yoshino et al., 2007). It is thought that only some strains may have the capacity to cause disease and differences between strains in the ability to cause localized or systemic infections have been demonstrated in animal models (Griffen et al., 1999).

Using multilocus sequence typing (MLST) it has been shown that up to eight sequence types of *P. gingialis* can be found in individual pockets at diseased sites in patients with chronic periodontitis (Enersen et al., 2008). Similarly, heteroduplex analysis of the ribosomal operon intergenic spacer region of *P. gingivalis* identified in subgingival plaque samples found 39% of these samples had multiple heteroduplex types (Igboin et al., 2009). At present very little is known about the genetic diversity and underlying population structure of *P. gingivalis*, the stability of strains within an individual over time or during disease development, or the transmission of *P. gingivalis* between individuals. *P. gingivalis* is naturally competent and able to exchange alleles between strains (Tribble et al., 2012; Kerr et al., 2014). Multilocus sequence typing indicates that there is frequent horizontal gene transfer and recombination (Frandsen et al., 2001; Koehler et al., 2003; Enersen et al., 2008; Enersen, 2011). To better understand the virulence and evolution of the bacterium a greater knowledge of its genetic diversity and genetic exchange is required. Genetic diversity, especially in genes encoding surface virulence factors can make a targeted immunotherapy more challenging.

Here we report the comparison of the genomes of 21 *P. gingivalis* strains sourced from around the world over a 25 year time period. We determined that there is extensive genomic recombination with overall limited sequence diversity. Notably, a subset of genes encoding specific surface virulence factors have multiple alleles that through recombination have produced a large variety of combinations.

## Materials and methods

### Bacterial strains

Thirteen *P. gingivalis* strains were sequenced in this study. These included 11 *P. gingivalis* isolates 11A, 7B TORR, 381, A74A1-28, 13–1, Afr-5B1, 3A1, 3_3, 15–9, ATCC 49417, and 84–3 that were a kind gift from R. Page, Washington University, Seattle, WA, USA. All clinical samples in this group were obtained by paper point sampling of subgingival plaque found in periodontal pockets at least 6mm deep in subjects that had at least four 6mm deep pockets, gingival bleeding on probing, no previous periodontal treatment, no antibiotic use in the past 6 months and good systemic health. These isolates were obtained from periodontitis patients residing in the United States, Sudan, Romania and Norway (Ali et al., 1994a,b, 1996, 1997; Slakeski et al., 2002). *P. gingivalis* W50 was a kind gift from P. Marsh (University of Leeds, UK) and has been in the laboratory stocks of the Melbourne Dental School for 24 years. *P. gingivalis* YH522 was obtained from H. Yoshimoto (Kanagawa Dental College, Japan) and has been in the laboratory stocks of the Melbourne Dental School for 18 years.

The publicly available closed genomic sequences of *P. gingivalis* strains W83 (Nelson et al., 2003), ATCC 33277 (Naito et al., 2008) and TDC60 (Watanabe et al., 2011); six draft genomes (FO185, FO566, FO568, FO569, FO570, W4087) available from the Forsyth Institute, Boston, USA; and a draft genome of a Chinese strain SJD2 (Liu et al., 2014) were included in the analyses. Background information for each strain and the relevant accession numbers are shown in Table 1.

**Table 1:** *****P. gingivalis*** strains referred to in this study**.

***P. gingivalis* strain**	**Collection date**	**Country**	**Isolation_source**	**INSDC Accession**
3_3	1995	USA	Subgingival plaque	PRJEB10280
3A1	1994	Norway	Subgingival plaque, from a ≥ 6 mm periodontal pocket	PRJEB10280
7BTORR	1995	USA	Subgingival plaque	PRJEB10280
11A	1996	Romania	Subgingival plaque, from a ≥ 6 mm periodontal pocket	PRJEB10280
13_1	1994	Sudan	Subgingival plaque, from a ≥ 6 mm periodontal pocket	PRJEB10280
15_9	1996	Romania	Subgingival plaque, from a ≥ 6 mm periodontal pocket	PRJEB10280
84_3	1994	Sudan	Subgingival plaque, from a ≥ 6 mm periodontal pocket	PRJEB10280
ATCC 49417	1987	Canada	Periodontal pocket	PRJEB10280
A7A1_28	1985	USA	Subgingival plaque, from a ≥ 9 mm periodontal pocket	PRJEB10280
AFR5B1	1994	Sudan	Subgingival plaque, from a ≥ 6 mm periodontal pocket	PRJEB10280
YH522	1997	Japan		PRJEB10280
W50	1958	Germany		PRJEB10280
381	1975	USA	Base of deep periodontal pocket	PRJEB10280
W83	1958	Germany		NC_002950.2
ATCC 33277^*^	1978	USA	Base of deep periodontal pocket	NC_010729.1
TDC60	2011	Japan	Severe periodontal lesion	NC_015571.1
SJD2	2009	China	Subgingival dental plaque	ASYL00000000
F0185			Subgingival plaque	AWVC00000000
F0566			Subgingival plaque	AWVD00000000
F0568	1983	USA	Subgingival plaque	AWUU00000000
F0569	1984	USA	Subgingival plaque	AWUV01000000
F0570	1984	USA	Subgingival plaque	AWUW00000000
W4087				AWVE00000000
*P. gulae* ATCC 51700	2001	Canada	Wolf gingival sulcus	ARJN00000000

* *Strain ATCC 33277 is now believed to be a streptomycin resistant mutant of strain 381 (Loos et al., 1990)*.

There were only 20 single nucleotide polymorphisms (SNPs) between the genome sequence of strain W50 from this study and the previously sequenced strain W83. These two isolates were collected at the same time from the same patient and presumably are the same strain. As a result, only W83 data are presented in these analyses to avoid bias.

Only a small number of SNPs were detected between strain (FDC) 381, sequenced in this study, and the genome sequence of ATCC 33277 (Naito et al., 2008) so only ATCC 33277 data is presented in this study. The recently published genome sequences of 12 *P. gulae* strains (Coil et al., 2015) were included in this study to provide a reference and phylogenetic outgroup. The Loup 1 *P. gulae* type strain (ATCC 51700) (Fournier et al., 2001) was obtained from the American Type Culture Collection.

### Preparation of DNA and sequencing

*P. gingivalis* strains and *P. gulae* were grown to stationary phase in Brain Heart Infusion medium supplemented with 0.5 g/L cysteine and 1 mg/L menadione. Cells were harvested by centrifugation and washed in 10 mM Tris-HCl pH 8.0. DNA was extracted using the Blood and Tissue Genomic DNA Isolation Kit (Qiagen) and concentrated to 100 ng/μL using ethanol precipitation then resuspended in 10 mM Tris-HCl.

Ilumina Nextera-based whole-genome sequencing producing 2 × 100 bp paired-end reads with an average insert size of ~500 bp was performed at The Australian Genome Research Facility (AGRF) using the Illumina HiSeq-2000 Platform with the CASAVA1.8.2 pipeline.

### Assembly and annotation of genomes

Assembly and annotation of sequences was performed at the Victorian Life Sciences Computation Initiative—Life Science Computation Centre. Contigs were created by *de novo* assembly using Velvet v1.2.07 (Zerbino and Birney, 2008) in conjunction with VelvetOptimiser v2.2.5 (https://github.com/tseemann/VelvetOptimiser). The assembled sequences were annotated using Prokka v1.11 (Seemann, 2014).

Manual curation of the annotation to remove false overlapping genes and correct translation initiation start sites was performed using eCAMBER (Wozniak et al., 2014). Genes encoding hypothetical proteins < 61 aa in length, with no similar proteins in the NCBI databases and lacking any detectable conserved domains, were omitted (Salzberg et al., 1998). This conservative approach resulted in the exclusion of only three genes in the *P. gingivalis* W83 genome of 1822 genes. The genome of *P. gulae* ATCC 51700 was included as a more phylogenetically distant outgroup to improve structural annotation predictions. InterProScan was used from within Geneious v 8.1.3 (Biomatters Ltd.) to identify signal peptides and other protein domains signatures for genes predicted to encode gingipains. Type IX secretion system C-Terminal Domain (CTD) signal sequences were identified by alignment (Veith et al., 2013). Gene identifiers used throughout this manuscript refer to those from the first published genome of *P. gingivalis* W83 (Nelson et al., 2003) as commonly used in the literature. For the updated NC_002950.2 version of the W83 genome these “PGXXXX” identifiers are referred to under the old_locus_tag qualifier.

### Phylogeny

The MLST gene data set used was the complete gene sequences from six of the seven genes (*ftsQ, gpdxJ, mcmA, pepO, pga, recA*) defined in the PubMLST *P. gingivalis* MLST Database http://pubmlst.org/pgingivalis/ (Jolley and Maiden, 2010). The *hagB* gene is a repetitive sequence and was not included in the phylogenetic analysis (as it is paralogous to *hagC*). Sequence manipulations were performed in Geneious v 8.1.3. Individual genes were codon aligned with MAFFT v 1.3.5 (Katoh and Standley, 2013) prior to testing several methods to generate phylogenetic trees. Inconsistent results in generating trees (and poor branch support) indicated that a bifurcating tree was unlikely to accurately represent the ancestry of these strains. Therefore, the MLST alignment was plotted as a NeighborNet network using SplitsTree4 (v 4.13.1) (Huson and Bryant, 2006). SplitsTree4 was used to calculate the δ score and Q-residual score as measures of “treelikeness” of the phylogenetic distance data (Holland et al., 2002). The Phi test implemented in Splits Tree4 was used to infer the likelihood of recombination in the DNA sequence alignment data.

Whole genome SNP phylogeny was computed for the *P. gingivalis* genomes with and without *P. gulae* using Parsnp v 1.0 (Treangen et al., 2014) with recombination detection enabled. Alignments without *P. gulae* were used to examine the phylogenetic relationship among the *P. gingivalis* strains. Phylogenetic trees were generated with FastTree (v 2.1.7) (Price et al., 2010) using the GTR evolutionary model and reliability tested with the Shimodaira-Hasegawa test for 2000 resampling steps. Consensus trees were calculated for branches with >75% support using a greedy clustering method. FASTA alignments were converted from the XML files generated by Parsnp using a custom script for analysis in SplitsTree4 as described above.

### Selection analysis

Selection analysis was conducted on codon aligned catalytic domains from proteins Kgp, RgpA, and RgpB. Positional references denote the location of the codon in the intact gene rather than the extracted catalytic domain sequence. The two variants of Kgp necessitated that selection analysis be conducted in each variant individually. Nucleotide diversity calculations (π) were performed with DNASP ver 5.10 (Librado and Rozas, 2009). In order to determine if codons in the catalytic domain were subjected to positive or negative selection, several methods within the HyPhy analysis suite were used (Pond et al., 2005). A conservative approach was taken for interpreting the results requiring that more than one method detected selection and that *P*-values or posterior probabilities were below 0.05 or 0.9, respectively. HyPhy analysis methods were implemented in the Datamonkey.org web portal (Pond and Frost, 2005). Methods tested included single likelihood ancestor counting method (SLAC; Kosakovsky Pond and Frost, 2005), fixed effects likelihood method (FEL, iFEL; Pond et al., 2006), and the fast unconstrained Bayesian approximation method (FUBAR; Murrell et al., 2013). All alignments were examined for signals indicative of recombination with GARD (Kosakovsky Pond et al., 2006) and the analysis adjusted accordingly.

### Sequence analysis

Average nucleotide identity (ANI) between strains was calculated as OrthoANI values calculated from the Orthologous Average Nucleotide Identity Tool, v 0.90 (Lee et al., 2016). Sequence alignments and manipulations were performed essentially as described above. The E-INS-I algorithm was used with a BLOSUM62 scoring matrix with a gap opening penalty of 1.03. Alignments of multiple gene regions were performed using the Geneious alignment tool using a global alignment with free end gaps, a 65% similarity cost matrix, a gap opening penalty of 12 and extension penalty of 3. To manually find and verify genes of interest across all 21 *P. gingivalis* genomes, a local database of genome nucleotide sequences was created within Geneious and the MegaBLAST search algorithm was used with default parameters to find and visualize hits.

### Assembly of gingipain and hagA genes

The repeat regions within the *hagA, rgpA*, and *kgp* genes were unable to be resolved by using the Velvet assembler, resulting in contig breaks within each of these genes. The *hagA, rgpA*, and *kgp* genes and their surrounding regions in the three closed genomes were compared using global and local sequence alignments within Geneious. Extensive manual curation was performed to identify and annotate unique and repeat regions within and across these genes and identify any conservation of gene order of the surrounding genes. These unique regions were used to identify contigs that could be assigned to a specific gene. The connections extending out from these contigs were then visualized using Bandage v 0.8.0 (Wick et al., 2015) and connected contigs compared to the reference genomes using MegaBLAST and annotated. A plausible path for scaffolding contigs was manually determined based on the order of the specific regions found in the three closed genomes and the depth of coverage of each of the contigs.

### Validation of Kgp assemblies

Predicted *kgp* assemblies from the 11 *P. gingivalis* strains and single *P. gulae* strain sequenced in this study were validated using Sanger sequencing. Primers were designed using Primer3 v 2.3.4 (Untergasser et al., 2007). Strain specific primer sets were designed to amplify 500 bp regions and repeat regions co-ordinates were used to exclude target sites (Table 2). Primers *kgp_F1* and *kgp_Rii* were used to produce a 5.2 kb amplicon for each strain, which was quantified and used as the template with each of the primers listed for capillary electrophoresis sequencing at The Melbourne Translational Genomics Platform.

**Table 2:** **Primers used for validation of ***P. gingivalis*** and ***P. gulae kgp*** assemblies**.

**Primer name**	**Sequence**	**Strains**
kgp_F1	ATTATTATTGCTGATCGCGGC	All
kgp_F2	TYATGCCRCATCAACCCTCT	All
kgp_F3	GGAACRACMAACGCCTCT	All
kgp_F4	CRGCGCATGGATCTGAGAC	All
kgp_F5	GYGATGGYTCGGTTATGCC	All except *P. gulae*
kgp_F6	TGCCAACGAAGCCAAGGT	All except *P. gulae*
kgp_F7	CGGTGTAGCTGCAGGCAA	All except *P. gulae*
kgp_F8	ACTTTCTGGGTATGCGCACA	All except *P. gulae*
kgp_F9	ACAGGCGCAACGAAGGTA	All except *P. gulae*
kgp_F11_A	CTGCAGCCGACTTCGAAG	7BTORR, 3A1, 84_3,49417, AFR5B1
kgp_F11_W	ACGCTTTGTTGGAAGAAGTGC	11A, 15_9, YH522
kgp_Rii	AGCRAGTTTYTCTACGTAAG	All except *P. gulae*
PgulK5f	CGCGCCGAATTGCTTAATGA	*P. gulae*
PgulK5f2	GTGTACTCACAGGGTGGAGC	*P. gulae*
PgulK5r1	GCTCCACCCTGTGAGTACAC	*P. gulae*
PgulK5r2	TCAAAGTCAGATGCTGCCGT	*P. gulae*
PgulK5r3	TCATTAAGCAATTCGGCGCG	*P. gulae*

### Pangenome estimation and visualisation

A visual overview of the relationship of each of these genome sequences to that of W83 was produced using BLAST Ring Image Generator, BRIG v 0.95 (Alikhan et al., 2011). Island Viewer v 3.0 was used to identify genomic islands in the *P. gingivalis* W83 genome (Dhillon et al., 2015).

Pangenome analysis was based on protein coding sequences only. Clustering of genes annotated as Coding DNA Sequences (CDSs) was performed using eCAMBER (Wozniak et al., 2014) to determine homologs. The parameter specifying the minimum percentage identity (PID) required for a match to be accepted was set at 50% and the default *e*-value score parameter was used.

### Lys-specific proteolytic activity

Whole cell Lys-specific proteolytic activity of *P. gingivalis* strains was determined using the chromogenic substrate N-(p-tosyl)-Gly-Pro-Lys 4-nitroanilide acetate salt (GPK-NA) as previously published (Toh et al., 2011).

### Modeling of Kgp adhesins

Modeling of the *P. gingivalis* K4 domain based on the known structure of the cleaved adhesin domain K3 (PDB: 3m1h) was performed using *FUGUE* (Shi et al., 2001) to generate a structural alignment, followed by *ORCHESTRAR* (Tripos International) to build a molecular model based on that alignment. Model quality was tested using the *align_all* algorithm from *PyMOL* (Schrodinger Inc.) to calculate the root-mean square deviation between structurally conserved atoms in the different CAD domains. Graphical representations of the CADs were generated using *PyMOL*.

## Results

### Phylogeny

The genomes of 13 *P. gingivalis* strains were sequenced and these data combined with a further 10 complete or draft genomes deposited in GenBank to provide an analysis data set (Table 1). The draft genome assemblies of the *P. gingivalis* strains ranged in size from 2,210,297 bp (W4087) to 2,424,225 bp (ATCC 49417) (Table 3). All *P. gingivalis* strains were closely related, with ANI values of orthologous fragment pairs between each of the 21 strains in the range 98.09–99.44%. In contrast pairwise comparison of each of the 12 published strains of *P. gulae* (Coil et al., 2015) with each of the *P. gingivalis* strains showed ANI values of between 91.88 and 92.77%, clearly indicating they are two distinct species, with values well below the 95–96% cutoff for species demarcation (Lee et al., 2016).

**Table 3:** *****P. gingivalis*** and ***P. gulae*** chromosomal contig assembly and annotation data**.

**Source**	**Strain**	**Ave insert size (nt)**	**Paired reads**	**Data Yield (Gb)**	**No. of contigs/scaffolds**	**Genome size (bp)^1^**	**GC content (%)**	**No of CDS^2^**
This study	49417	486	7,318,812	1.46	77	2,424,225	48.4	1925
	11A	452	7,599,986	1.52	89	2,304,118	48.4	1800
	13_1	448	7,180,084	1.44	69	2,341,110	48.3	1829
	15_9	405	7,192,577	1.44	68	2,252,483	48.4	1751
	3_3	463	9,240,096	1.85	72	2,312,663	48.3	1801
	3A1	459	6,997,858	1.4	56	2,343,280	48.3	1849
	7BTORR	469	6,889,800	1.38	72	2,248,982	48.4	1764
	84_3	439	6,709,468	1.34	50	2,325,183	48.4	1832
	A7A1_28	473	9,863,293	1.97	22	2,222,676	48.6	1738
	AFR5B1	493	9,005,662	1.8	88	2,290,524	48.6	1798
	YH522	466	7,159,737	1.43	53	2,257,351	48.5	1758
	*P. gulae*	492	9,430,272	1.89	92	2,323,774	50.4	1852
Genbank draft genomes	SJD2				117	2,339,271	48.4	1875
	F0185				137	2,236,685	48.6	1759
	F0566				189	2,282,374	48.4	1774
	F0568				166	2,315,008	48.4	1825
	F0569				136	2,236,098	48.5	1757
	F0570				135	2,266,638	48.5	1810
	W4087				125	2,210,297	48.5	1749
Genbank completed genomes	W83				1	2,343,476	48.3	1819
	33277				1	2,354,886	48.4	1830
	TDC60				1	2,339,898	48.3	1822

*No plasmids have been discovered in P. gingivalis to date*.

1 *For draft genomes, size is the total length of the contigs*.

2 CDS numbers as determined in this study by manual curation.

An alignment derived from a concatenated set of MLST genes was examined using a NeighborNet network. The resultant network generated with uncorrected nucleotide distances was not tree-like displaying a high level of reticulation (δ score = 0.4196, Q-residual score = 0.02411; Figure S1). A Phi test for recombination suggested a high likelihood of recombination (p < 0.0001) among the selected MLST genes used for this analysis. These results are consistent with Koehler's previous MLST study that found a high rate of recombination (Koehler et al., 2003). The discriminatory power of the MLST method is entirely dependent on the selection of appropriate housekeeping genes. P. gingivalis genes including nah were excluded from this scheme by previous studies due to their limited variation (Enersen, 2011). The official MLST typing scheme for P. gingivalis (http://pubmlst.org/pgingivalis) still includes the hagB gene despite this being a paralog with two nearly identical copies in the W83 strain. This shows that typing systems can be inaccurate without genome wide information from a broad selection of strains before developing such systems.

**Figure 1:**
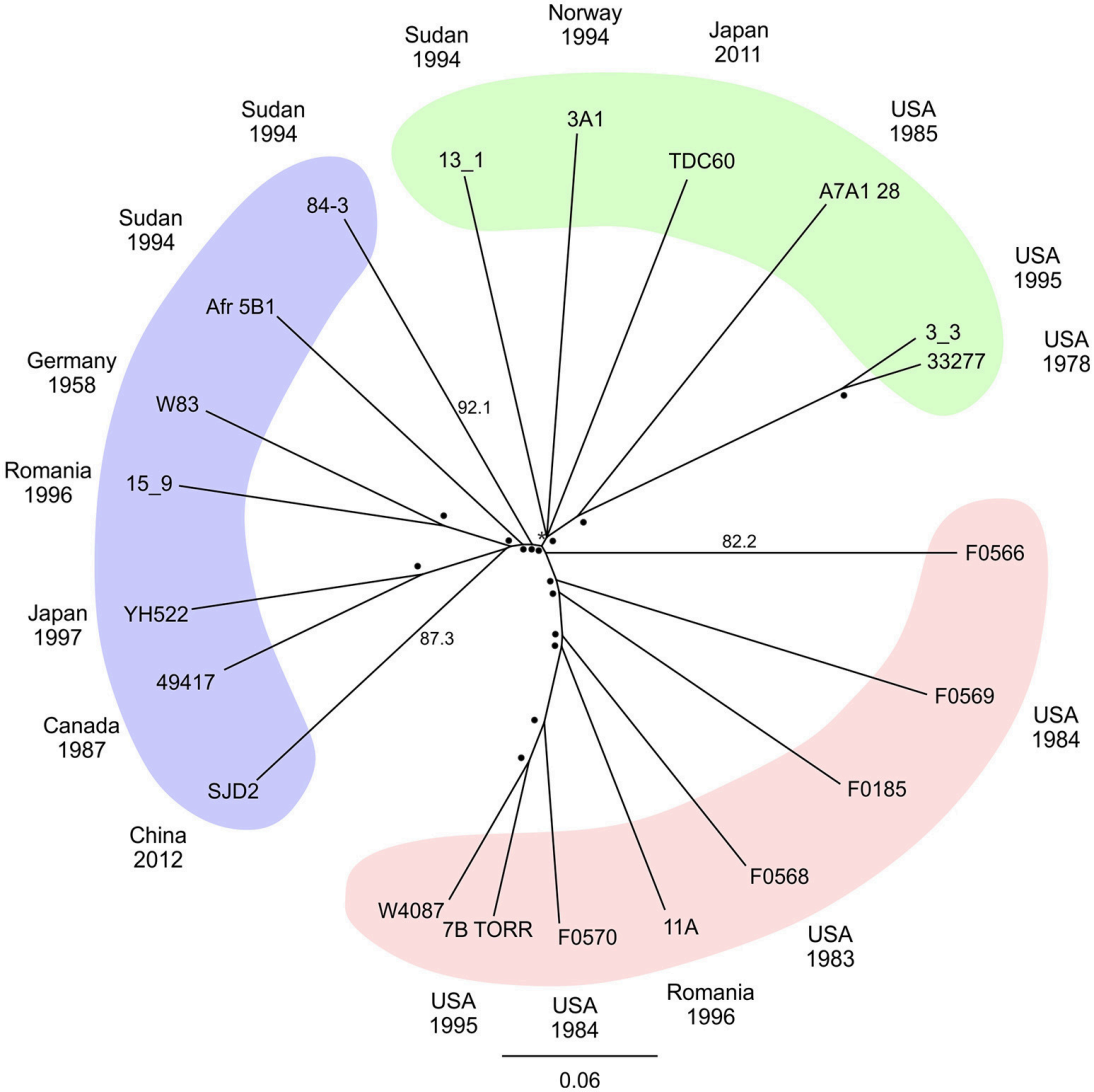
**Radial phylogenetic tree**. Unrooted approximate maximum likelihood consensus tree of whole genome P. gingivalis core SNP alignment. Plotted with percentage support values determined by Shimodiara-Hasegawa resampling test placed at the node or up the branch to improve readability, values of 99–100% are depicted by (•) with lower values written. Colored areas highlight major groupings of the taxa. The date and country of isolation of each strain where known are shown in the outer ring of the diagram.

Due to the limitations of the MLST method a SNP based approach was adopted using whole genome data. The P. gingivalis SNP alignment was subjected to approximate maximum likelihood analysis to yield an unrooted tree with strains separated into three broad clades (Figure 1). Resampling branch support was high for most branch points but three strains (13_1, 3A1 and TDC60) could not be clearly resolved resulting in a polytomy. A NeighborNet network derived from the whole genome SNP alignment yielded a more tree-like network than that MLST data set (δ score = 0.251, Q-residual score = 0.000533; Figure S2) confirming that a bifurcating tree adequately describes the phylogeny of these organisms.

### Pangenome

**Figure 2:**
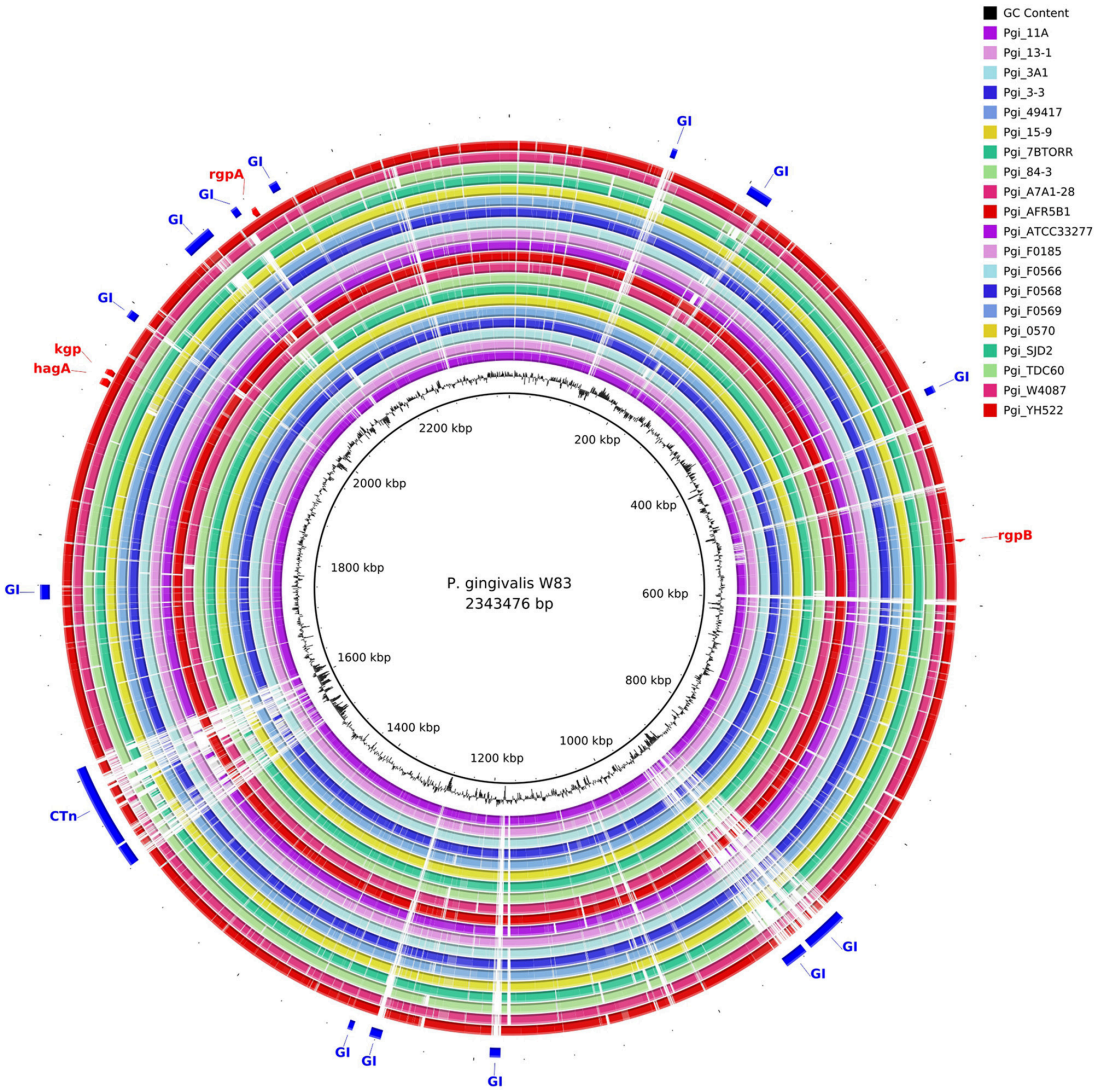
**Comparison of ***P. gingivalis*** genomes by BLAST Ring Image Generator (BRIG)**. The contigs of 20 P. gingivalis genomes are compared with the complete nucleotide sequence of strain W83 as reference. There are two areas of high divergence. P. gingivalis W83 contains several predicted genomic islands, many of which are highly divergent between strains. The genomic island centered on 900 kbp is bounded by a series of repeats, integrases and a tRNA. It is likely that this genomic island has been built up by multiple insertion events given the large number of tandem integrases in this region. A highly variable region centered around 1550 kbp contains a putative conjugative transposon (CTn).

The gene content of the P. gingivalis W83 strain was highly conserved in the 20 other strains as seen in the BRIG diagram (Figure 2). There are two major regions of sequence variability, a genomic island of ~70 kbp centered around 900 kbp on the chromosome and a homolog of the Bacteroides conjugative transposon CTnDot centered around 1550 bp. The predicted genome size of the ATCC 49417 strain was 70 kb larger than any of the other genomes (Table 3) and two relatively complete prophage genomes were detected in this strain, one of which was also present and highly conserved in the AFR5B1 strain (Figure S3). Although numerous genome rearrangements and different integration sites for insertion elements have been observed between the three strains with complete genomes, it is problematic to determine these sorts of changes with fragmented draft genomes, so our study has focused on a comparison of the predicted protein coding regions. A total of 3740 protein-coding gene clusters were found across the 21 P. gingivalis strains and 1488 high-frequency genes were present in 17 or more of the strains (Data Set S1). Of the remaining gene clusters, 345 were medium-frequency genes present in 6–16 of the P. gingivalis strains whilst 1907 were low-frequency genes present in five or less strains. The majority of gene clusters occurred only once per strain, but 145 contained paralogs, with two or more copies in at least one strain.

### Gingipain catalytic domains

Gingipain Kgp catalytic domain crystal structure shows a caspase-like fold where catalysis occurs, followed by an immunoglobulin-like fold (de Diego et al., 2014; Gorman et al., 2015). The Kgp structure reveals a flat substrate binding site that would be amenable to interactions with a wide array of proteins. Cleavage site specificity is produced by the constraints of the S1 binding pocket into which the side chain of Lys residue is inserted. The RgpB catalytic domain has a structure similar to Kgp_cat_ (Eichinger et al., 1999).

**Figure 3:**
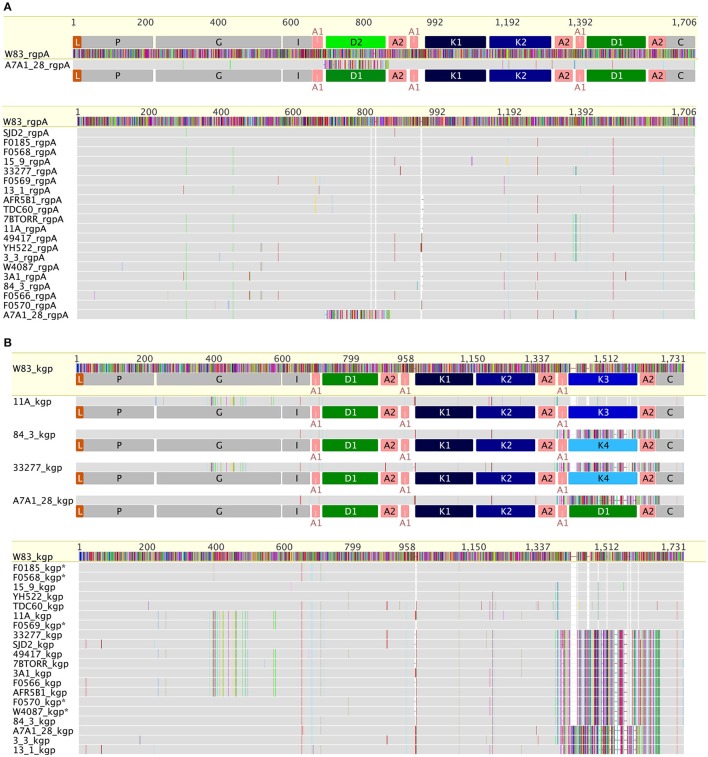
**Comparison of RgpA (A)** and Kgp **(B)** variability across the P. gingivalis strains. RgpA and Kgp are comprised of a leader peptide (L), a propeptide region (P), a catalytic domain (G) of around 360 amino acids, an immunoglobulin fold (I) followed by three or more of modular adhesin domains comprised of CADs (K1–K4) and DUF2436 domains (D1–D2) and a conserved C-terminal domain (C). Kgp separates into four groups based on specific substitutions in the catalytic domain combined with differences in the C-terminal CAD. The specific substitutions in the Kgp catalytic domains around the catalytic site that may impact catalysis, substrate binding or propeptide interactions are discussed in the text. In contrast to Kgp in RgpA there is a high level of conservation of the catalytic domain sequence and the CADs with only strain A7A1_28 showing a substituted D1 domain.

RgpA and RgpB catalytic domain sequences were highly conserved across all of the strains, with only 14 substitutions across the 346 amino acids of the RgpA_cat_ domain in the context of overall nucleotide diversity of π = 0.0109 (Figure 3A). There were two positions in the RgpA_cat_ domain that had specific conservative substitutions in approximately half of the strains, Ile302Val and Val433Ala. The remainder of substitutions occurred in a low number of strains and appeared to be random. The RgpA propeptide was highly conserved across all strains with only two substitutions in single strains relative to the W83 sequence. An analysis to detect signals of evolutionary selection using the 17 unique RgpA_cat_ domains determined that RgpA_cat_ domain had seven codon positions under negative selection and none under positive selection (Table S1). Similarly, the RgpB_cat_ domain was highly conserved with a nucleotide diversity level of π = 0.01955 across 20 unique sequences. Selection analysis detected 10 codons under negative selection and none under positive selection (Table S1). There were significant differences between the RgpA and RgpB propeptides (43 substitutions and 3 indels across 199 amino acids) indicating that they have distinct binding sites or other constraints on sequence variation.

**Figure 4:**
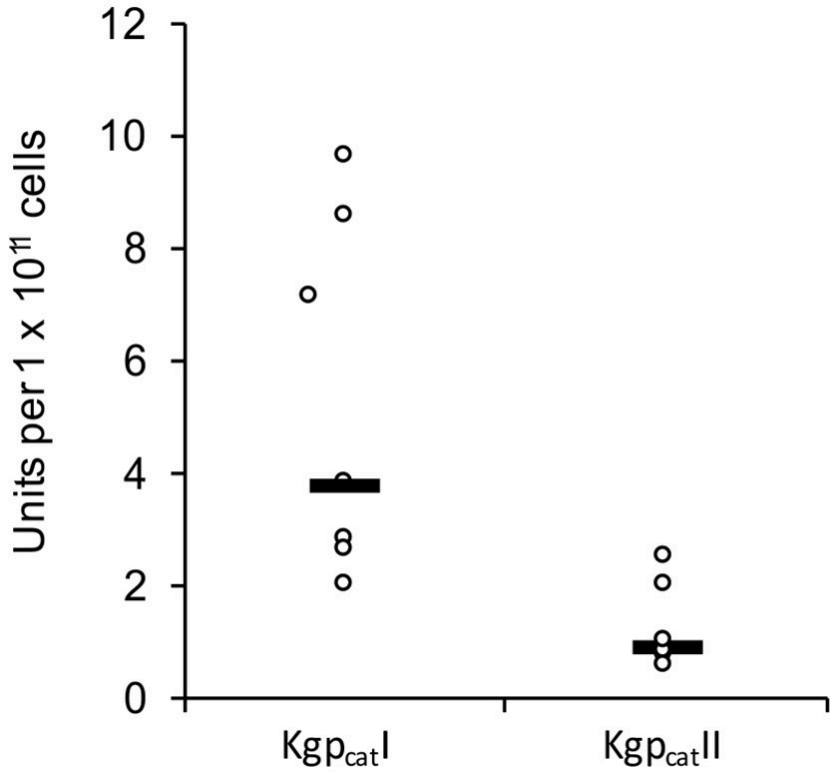
**Whole culture Lys-specific proteolytic activity of 12 ***P. gingivalis*** strains determined using the chromogenic substrate N-(p-tosyl)-Gly-Pro-Lys 4-nitroanilide acetate salt (GPK-NA) as previously described (Toh et al., 2011)**.

In constrast there were two distinct variants of the catalytic domain of Lys-gingipain (Kgp_cat_) with 21 specific amino acid substitutions in a 150 amino acid region that encompasses the Lys-gingipain catalytic site (Figure 3B). We refer to these variants as Kgp_cat_I and Kgp_cat_II. Mapping these substitutions to the known structure of Kgp_cat_ (Gorman et al., 2015) showed that five of the variable residues occur in proximity to the active site pocket and 11 form an adjacent contiguous surface exposed patch (see Section Discussion). Examination of both Kgp_cat_ variants for selection signals indicated that of the eight unique Kgp_cat_I and five unique Kgp_cat_II domain sequences, three and one codon were under negative selection respectively and no codons were detected to be under positive selection (Table S1). Determination of whole cell Lys-X proteolytic activity revealed that those strains that harbored Kgp_cat_I had on average higher activity than those strains harboring Kgp_cat_II (Figure 4).

### Haemagglutinin-like adhesins

Due to identical repeat sequences within and between the regions of the hagA, rgpA, and kgp genes encoding adhesins (CADs; IPR011628) these regions could not be fully resolved using automated assembly. Therefore, extensive manual assembly was performed with reference to the three closed genomes to identify contigs that could be assigned to a specific gene. The rgpA and kgp CAD encoding regions were assembled and the kgp assemblies for each strain were validated by Sanger sequencing (Table 2). Unfortunately, the highly repetitive nature of hagA and the length of the repeated units confounded assembly of hagA from draft genomes.

The CAD adhesins of Kgp of strain W83 are known to occur in the order K1-K2-K3. N-terminal to K1 is another domain module DUF2436 (conserved Pfam Domain of Unknown Function; IPR018832) (D1) giving the adhesin organization D1-K1-K2-K3. Defining a CAD or DUF2436-like domain subtype as having >90% amino acid identity indicates that in other *P. gingivalis* strains, following Kgp_cat_ are also modules D1-K1-K2. However, the C-terminal domain varies, being an additional copy of D1 or a fourth CAD, K4 (Figure 3B). C-terminal to RgpA_cat_ the CAD and DUF2436-like domains occur in the order D2-K1-K2-D1, with the exception of strain A7A1-28 which has D1-K1-K2-D1 (Figure 3A). This shows that there is combinatorial modularity in the adhesin regions of the gingipains. Similarly CAD and DUF2436 domain modularity is evident in *P. gulae* (Figures 3A,B).

**Figure 5:**
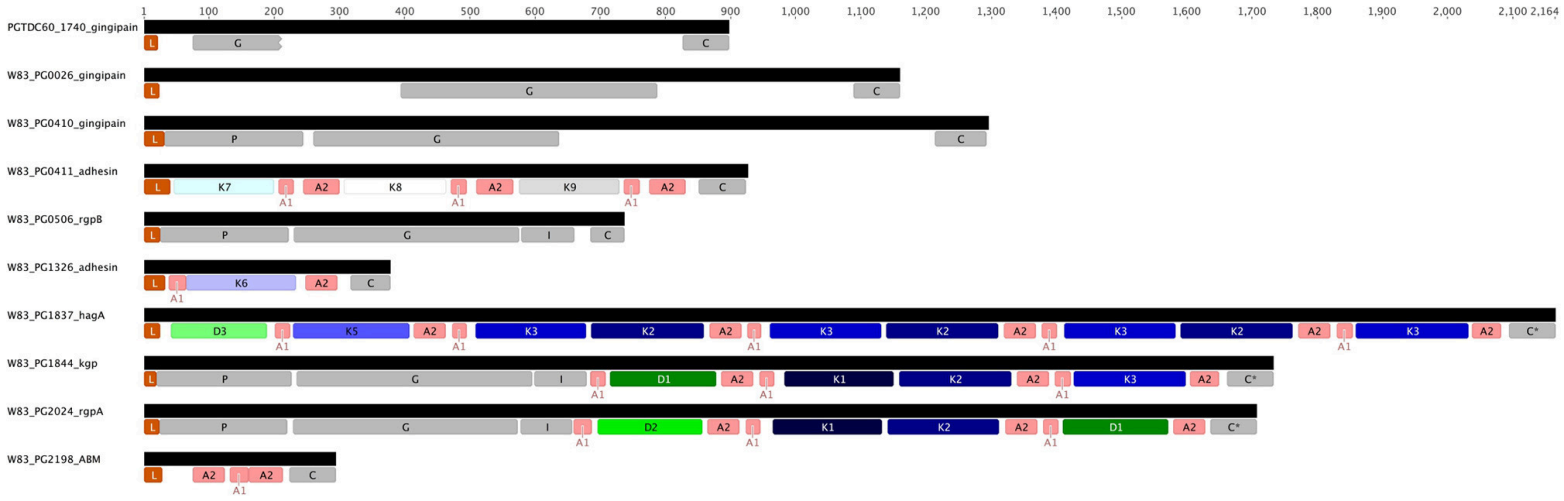
**Structure of the genes encoding known and putative gingipains and modular adhesins containing CADs**. The gingipains are comprised of a leader peptide (L) that is cleaved during translocation across the inner membrane, an unusually long propeptide region (P; InterPro Accession number IPR012600) of ~200 amino acids and a catalytic domain (G; IPR001769) of around 360 amino acids that is followed by an immunoglobulin fold (I; IPR005536). RgpA and Kgp contain three or more modular adhesin domains comprised of CADs (K1–K3; IPR011628) and DUF2436 domains (D1-D2; IPR018832). There is a conserved C-terminal domain (C; IPR026444) of ~80 amino acids. HagA lacks a proteolytic domain and cognate propeptide. Interspersed between and flanking the immunoglobulin fold and modular adhesins are adhesin binding motifs (A1–A2).

Examination of HagA encoded in the completed W83 genome shows further DUF2436 and CAD domains, D3 and K5, respectively, which are followed by repeated K3-K2 pairs and an orphan C-terminal K3 (Figure 5). In contrast, strains TDC60 and ATCC 33277 do not have the orphan K3 domain and ATCC 33277 has an additional K3-K2 repeat.

Interestingly, the variants of the *P. gingivalis* Kgp CADs are not linked to the variants of Kgp_cat_ (Table 4), which divides the Kgps into five discernible groups. These Kgp groupings are incongruent with the clades identified by genomic SNP-based analysis. Kgp phylogenetic analysis indicated a high level of recombination (Figure S4).

**Table 4:** **Distribution of variable surface proteins across P. gingivalis strains**.

**Strain and phylogenetic clade^1^**	**Kgp catalytic domain^2^**	**Kgp terminal adhesin**	**FimA**	**Accessory Fimbriae (FimCDE)**	**MfaI**	**RagAB**	**Tpr**	**PrtT catalytic domain**
W83	I	K3	IV	I	Disrupted	1	I	I
15_9	I	K3	IV	I	II	1	I	I
SJD2	II	K4	I	I	II	3	I	II
49417	II	K4	III	II	II	1	I	I
YH522	I	K3	IV	I	I	3	I	I
AFR5B1	II	K4	I	II	II	2	I	II
84_3	I	K4	I	II	II	1	I	I
33277	II	K4	I	II	I	4	Absent	II
3_3	I	D1	I	II	I	4	II	Disrupted
A7A1_28	I	D1	II	II	II	3	III	I
3A1	II	K4	II	II	I	4	III	II
TDC60	I	K3	II	II	I	4	Disrupted	I
13_1	I	D1	II	II	I	4	II	I
F0566	II	K4	I	II	I	3	I	II
F0569	II	K3	II	II	I	4	II	II
F0185	I	K3	II	II	I	2	II	I
F0568	I	K3	II	II	I	2	III	I
11A	II	K3	II	II	II	2	II	I
F0570	I	K4	I	II	I	2	I	I
7BTORR	II	K4	II	II	II	2	III	Disrupted
W4087	I	K4	II	II	I	2	III	I

1 *Coloring matches the phylogenetic classification shown in Figure 1*.

2 *In P. gingivalis strain W83 Kgp = PG1844; FimA = PG2132; MfaI = PG0176; RagAB = PG0185/PG0186; Tpr = PG1055; PrtT = PG1548*.

PG0411, in a similar manner to HagA, is composed of adhesin domains and has an N-terminal Sec-dependent leader sequence and a type IX secretion system CTD signal sequence indicating it is exported to the cell surface and attached in the same manner as the other adhesin domain-containing proteins. Intriguingly in strain W83 the PG0410 gene is immediately upstream of PG0411 and encodes a gingipain proteinase that contains no adhesin domains but does have a type IX secretion system CTD sequence.

### Fimbriae

Four distinct types of *fimA* were detected across the 21 strains in this study, corresponding to types I, II, III, and IV although only one strain, ATCC 49417, contained a type III *fimA* gene and no type V genes were detected (Figure S5; Table 4). A network analysis showed the very high level of divergence between *fimA* types and obvious signs of horizontal gene transfer (Figure S6).

In addition to the highly divergent *fimA* types, a DNA alignment of the *fimA* region for all 21 strains revealed that the genes encoding the fimbrial accessory proteins (*fimCDE)* have two distinct types (Figure S7). This divergent region, encompassing the last 370 bp of the 3′ end of the *fimC* gene as well as entire *fimD* and *fimE* genes, is likely to result from insertion of variant DNA at a second recombination site within the *fimA* gene cluster. The two distinct groups present (*fimCDE* type I and II) have < 43% aa identity for FimD and FimE, compared to the >96% nt identity across all strains for the 5′ region of the *fimC* gene. *FimCDE* type I was associated with *fimA* type IV however this linkage can be disrupted as observed in strain SJD2, a recent clinical isolate from China, which has *fimCDE* type I genes paired with *fimA* type I.

Both known alleles of the minor fimbriae fibrillin gene *mfaI* (Nagano et al., 2015) were found in the sequenced *P. gingivalis* strains with type II occurring in eight of the strains (Table 4). It is worth noting that both of the commonly used laboratory strains W83 and the ATCC 32277 type strain appear to be atypical for fimbriae production. The *mfaI* gene of W83 has been disrupted by an insertion element, which was detected in both W83 and W50 genomes. In addition, as previously published, FimA expression is greatly decreased in strain W50 because of a mutation in the ATP-binding site of the FimS histidine sensor kinase that regulates this operon. This mutation results in premature termination of the FimS protein and is present in both W83 and W50 but not in any other strain. ATCC 33277 has a non-sense mutation in *fimB* that results in unusually long major fimbriae (FimA) and an increased level of detachment and secretion of these long fimbriae (Nagano et al., 2013). This point mutation was not found in any of the other sequenced strains, including the 381 strain that was thought to be the provenance of the ATCC 33277 type strain (Loos et al., 1990).

### Other variable surface proteins

All four known variants of the TonB-linked integral outer membrane protein RagA and associated lipoprotein RagB were detected in this study. RagAB Type 2 and type 4 were the most prevalent, being found in seven and six strains, respectively (Table 4).

At least two *P. gingivalis* streptopain homologs have been reported, PrtT (Kuramitsu, 1998) and periodontain (PG1427; Nelson, 1999). PrtT (PG1548) is an 841 amino acid long CTD family protein with a predicted C10 cysteine peptidase catalytic domain of 195 amino acids that is preceded by a predicted propeptide of 185 amino acids in length. Two distinct catalytic domain alleles of PrtT were detected in the current study that differ by 11 specific amino acid substitutions in an 18 amino acid region of the catalytic domain. Type I was the most prevalent allele being found in 13 strains, whilst two strains had no functional alleles (Table 4; Figure S8).

Three distinct variants of the gene encoding the 503 aa outer-membrane bound Thiol Protease (Tpr; PG1055) were found in 20 of the 21 *P. gingivalis* strains although the gene was disrupted in TDC60 (Table 4; Figure S9). The gene was not found in strain 33277.

## Discussion

*P. gingivalis* is a normal member of the oral microbiota and is widely considered to be a keystone pathogen in chronic periodontitis (Hajishengallis et al., 2012). Numerous attempts have been made to classify the bacterium into strains that are associated with disease (Griffen et al., 1999; Amano et al., 2004; Yoshino et al., 2007). The genomes of all 21 *P. gingivalis* strains examined in this study showed that global strains are closely related, with ANI values between strains of above 98%. The strains could be separated into three broad, shallow clades, and distribution of the strains between the clades was unrelated to the time and country of isolation (Figure 1).

*P. gingivalis* had a pangenome of 3740 gene clusters. Core genes are often defined as those that are present in all strains of a species. However, genes may not be detected in some strains due to errors in sequencing, assembly, gene calling, or through mutations arising from multiple passaging within the laboratory. Rather than defining core and variable/flexible genes, Cordero and Polz (2014) suggested categorizing gene occurrence as high, medium or low frequency, as these divisions are more accurate and can be linked to different evolutionary processes. Core metabolic and housekeeping functions are typically encoded by high frequency genes that are maintained through both vertical inheritance and horizontal recombination. The 1488 high-frequency genes detected in 17 or more *P. gingivalis* strains comprise 83% of the average genome size of 1803 predicted CDSs. This is comparable to the 1476 genes recently estimated by comparative genomic hybridization microarray analysis of seven *P. gingivalis* strains being representative of the seven capsular serotypes (Brunner et al., 2010). Generally the number of genes in the core genome slowly decreases as more genomes are added (Collins and Higgs, 2012). Low frequency genes are gained and lost at high rates from individual genomes and are often found as singletons. Negative frequency-dependent selection arising from interactions with the immune system or predation by bacteriophage can act to drive and stabilize diversity within a population and many low-frequency genes are thought to be under this type of selection (Cordero and Polz, 2014). For example, the PG0410 gingipain gene was detected in only two *P. gingivalis* strains (W83 and F0185), however an ortholog of this gene with 99.5% nucleotide identity was found in three of the 12 *P. gulae* strains in a syntenous location, suggesting the gene may be acquired through homologous recombination. The maintenance of this gene at low frequency in both species is indicative that negative frequency-dependent selection is occurring.

The surface of *P. gingivalis* is heavily decorated with ~32 proteins that are exported and attached by the Type IX secretion system (Sato et al., 2010; Veith et al., 2013) as well as a range of other proteins including fimbrial proteins, lipoproteins, and integral membrane proteins. A subset of these proteins comprises the major virulence factors of the bacterium. Antigenic variation is a strategy commonly employed by pathogens to avoid the human immune system. The ability to quickly alter antigens provides an obvious advantage to the pathogen.

**Figure 6:**
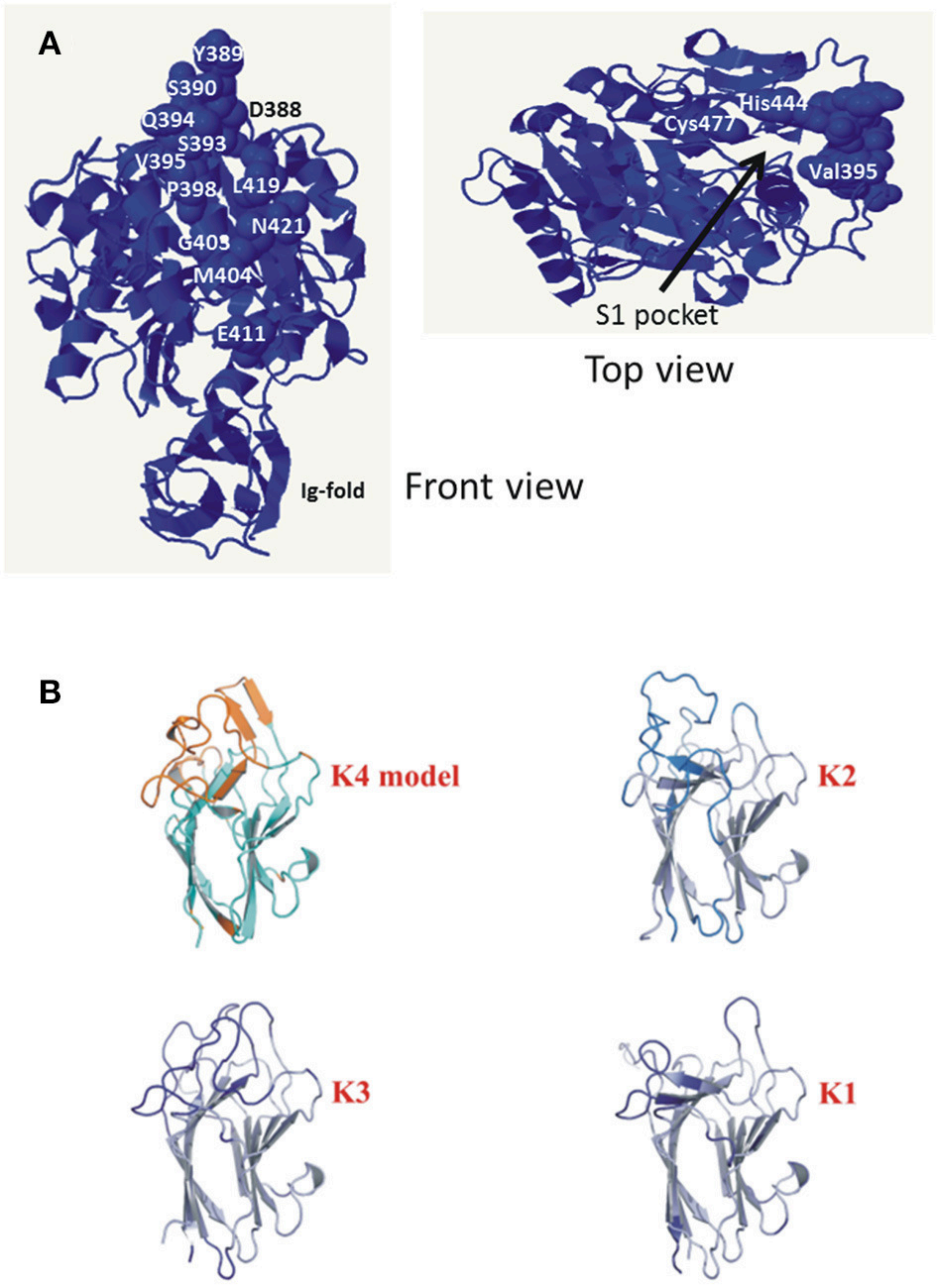
**(A)** Amino acid residues of Kgp_cat_I that vary in Kgp_cat_II. Front view shows the variable residues clustered on one face of the gingipain catalytic domain. The top view is a rotation of the front view ~90° to the right and ~90° down and shows the catalytic Cys477 and His444 relative to variable residue Val395. Structure rendered is 4TKX (Gorman et al., 2015). Modeling of the specific amino acid residue changes in Kgp_cat_II relative to Kgp_cat_I based on the known structure of *P. gingivalis* W50 Kgp_cat_I showing the surface-exposed nature of the residues (Gorman et al., 2015). **(B)** Comparison of the modeled K4 domain against the known structures of the cleaved adhesin domains of *P. gingivalis* Kgp.

Three gingipains, RgpA, Kgp, and RgpB, comprise the major *P. gingivalis* cell-surface associated proteinases. They are capable of degrading a variety of host proteins and dysregulating host defenses and are critical for bacterial colonization and the establishment of dysbiosis and disease (O'Brien-Simpson et al., 2001, 2005, 2009; Hajishengallis et al., 2012). The catalytic domains of the RgpA, Kgp, and RgpB gingipains that belong to the distinct cysteine proteinase clan C25 (Barrett and Rawlings, 2001), are found in all strains of *P. gingivalis* examined to date (Bhogal et al., 1997; Slakeski et al., 1998; Curtis et al., 1999a; Potempa and Nguyen, 2007). The *rgpA, kgp*, and *rgpB* gene products all contain a signal peptide of ~22 amino acids in length, an unusually long propeptide of over 200 amino acids in length, and a catalytic domain of ~480 amino acids. The RgpA and RgpB catalytic domains were nearly identical to each other and there was little variation across all *P. gingivalis* strains, whereas two distinct variants of the Kgp catalytic domain (Kgp_cat_I and Kgp_cat_II) were observed (Figure 3). These variants were not associated with phylogenetic clustering of the strains. Kgp_cat_I was present in 12 *P. gingivalis* strains including W50/W83 and TDC60 and Kgp_cat_II was present in nine strains including ATCC 33277 and ATCC 49417 (Table 4). The cell associated enzymatic activity of the Kgp_cat_II variant appeared to be lower than that of the Kgp_cat_I variant. Examination of the known crystal structure of the *P. gingivalis* W50 Kgp_cat_ domain, that consists of a central ten stranded β-sheet surrounded by ten α-helices forming an α*-*β sandwich (Gorman et al., 2015), revealed that five of these variable residues have at least one atom within 10 Angstrom of the catalytic site which may directly affect catalytic activity (Figure 6A). In addition, 11 of these residues (Tyr389Ser, Ser390Tyr, Ser393Pro, Gln394Lys, Val395Ile, Pro398Gln, Gly403Arg, Met404Val, Glu411Asp, Asn419Ser, and Leu421Pro) form a contiguous, surface exposed patch adjacent to the catalytic site that could alter protein:protein interactions with either another *P. gingivalis* surface protein (adhesin) or an exogenous protein (substrate; Figure 6A).

Closer examination of the catalytic domains for evolutionary selection detected negative selection acting on a number of amino acids in these regions. RgpA and RgpB had the greatest number of codons suggested to be under negative selection (8 and 10, respectively) while both Kgp variants had only three codons under negative selection. Interestingly, none of the amino acids involved in the surface exposed patch described above was demonstrated to be subject to positive selection as might be expected for such a region. The combination of a conservative analytic approach and the limited amount of nucleotide sequence diversity likely explains the low number of codons demonstrating signals for selection. Detection of only negative selection could be attributed to the fact that only the catalytic domain, which is likely to be under significant structural constraints, was examined.

The genes *rgpA* and *kgp* also encode four repetitive haemagglutinin-adhesin domains known as CADs that are located in the polypeptide region C-terminal to the catalytic domains (Figures 3, 5; O'Brien-Simpson et al., 2003; Li et al., 2010). Only the C-terminal CAD showed inter-strain variability with one of three variants present (Figure 3B). The crystal structures of three of the *P. gingivalis* W50/W83 Kgp CADs (K1, K2, K3) have recently been reported. The three CAD domains all have a conserved β-sheet core with structural differences limited to a series of solvent exposed loops (Li et al., 2010, 2011). The known crystal structure of *P. gingivalis* K3 (Li et al., 2011) was used to model the variant that we refer to as K4 and determine the location of the variable residues between K3 and K4. The differences between the K3 and K4 domains were restricted to the exposed surface loops—these are predicted to be structural differences as the sequences have inserts relative to each other (Figure 6B). Our modeling is consistent with these variable regions being the areas that interact with other molecules. The differences in exposed surface loops between CADs are proposed to affect the interaction of these CADs with other proteins and may potentially alter the host immune response to the bacterium or specificity of binding to host proteins.

RgpA, Kgp, and HagA also contain DUF2436 (conserved Pfam Domain of Unknown Function) domains that are similar in length (~160 aa) to the CAD domains and are similarly thought to be adhesins. These CAD and DUF2436 domains were found in five *P. gingivalis* proteins (Figure 5) and nine distinct types of CAD domains (K1–K9) and three types of DUF domains (D1–D3), based on amino acid sequence, were encoded in the *P. gingivalis* pangenome (Figure S10). Interspersed between and flanking CAD and DUF domains are adhesin binding motifs (ABM) ABM1 and ABM2 that may function in binding to host proteins and in assembly of the processed proteinase and adhesin domains into complexes at the cell surface (Slakeski et al., 1998; O'Brien-Simpson et al., 2003, 2005). Homologs of RgpA, RgpB, and Kgp are encoded in the *P. gulae* genome although there were significant differences with the *P. gingivalis* sequence (Lenzo et al., 2016; Figure 3).

The *P. gingivalis* major fimbrillin protein FimA, polymerises to produce long structures of up to 6 μm that protrude from the cell (61). FimA has been classified into five types (I–V) based on identified N-terminal amino acid sequences, nucleotide sequences and serological specificity (Lee et al., 1991; Fujiwara et al., 1993; Nakagawa et al., 2000). The *fimA* gene occurs within a seven gene cluster of *fimX, pgmA, fimA*-*fimE* (Frandsen et al., 2001). FimB modulates fimbrial length and FimC-E are accessory fimbrial proteins (Yoshimura, 1993; Watanabe et al., 1996; Nishiyama et al., 2007; Nagano et al., 2010). Based on a PCR typing system, type II has often been associated with disease and with increased virulence in laboratory studies (Amano et al., 1999; Fabrizi et al., 2013; Feng et al., 2014). However, studies using these PCR based typing systems have also shown that type II is the most widely distributed type among both healthy subjects as well as those with periodontitis (Moon et al., 2013). Four highly divergent types of *fimA*, corresponding to the known types I–IV were detected across the 21 strains in this study (Figure S4; Table 4). Two distinct forms of the FimC-E fimbrial accessory proteins that are thought to be localized to the tips of the fimbrial filaments and involved in host adhesion were also identified. Mutant strains devoid of accessory proteins have been shown to be less virulent in a mouse periodontitis model (Pierce et al., 2009). Kerr et al. (2014) demonstrated *fimA* transfer between *P. gingivalis* strains and also found transfer of the *fim*CDE region in some cases. The two known alleles of the minor fimbriae fimbrillin gene *mfaI* (Nagano et al., 2015) were found in the sequenced *P. gingivalis* strains. The Mfa minor fimbriae are important in interactions and biofilm formation with other oral bacteria (Park et al., 2005).

Several other surface proteins with variable alleles were detected that may also play a role in antigenic variation through gene conversion. RagAB are a TonB-linked integral outer membrane protein and associated lipoprotein that are reported to be involved in solute transport across the *P. gingivalis* outer membrane (Hanley et al., 1999; Goulas et al., 2016). They have been shown to form oligomeric complexes (Glew et al., 2014) and were originally described due to their immunogenicity in periodontal patients (Curtis et al., 1991, 1999b). Four distinct sequence types of RagAB have been described and linked to the virulence of the bacterium (Hall et al., 2005). These four sequence types were confirmed in this study with type 2 and type 4 being the most prevalent. Three distinct variants of the gene encoding the outer-membrane bound thiol protease Tpr were found in this study. Tpr is capable of degrading both complement proteins and the LL-37 antimicrobial peptide and may play a role in disruption of the immune system (Bourgeau et al., 1992; Staniec et al., 2015). Two distinct catalytic domain variants of the *P. gingivalis* streptopain homolog PrtT were also detected in this study.

In contrast to the variable surface virulence factors described above, Hbp35 (PG0616), LptO (PG0027), CPG70 (PG0232), integral outer membrane protein PG1823, peptidyl arginine deiminase (PPAD, PG1424) and P59 (PG2102) that are amongst the most abundant *P. gingivalis* surface proteins showed little variation between strains.

A comparison of the occurrence of all the variable surface proteins in the *P. gingivalis* strains formed a mosaic that was not related to the SNP based phylogeny (Table 4). In conjunction with the other surface proteins discussed above and assuming no genetic linkage of genes, the variability in these eight protein domains alone is enough to generate nearly 7000 different combinations. None of the 21 *P. gingivalis* strains in this study had the exact same combination of variants of these proteins. These variable surface proteins all had multiple variants present within *P. gingivalis* suggesting that the alleles encoding variants may be under negative frequency-dependent selection resulting in high rates of gene turnover, thus maintaining the diversity of these genes within the population. *P. gingivalis* is naturally competent, is not reported to contain plasmids, and is capable of gene conversion through homologous recombination, as well as possessing a suite of other mobile elements including a conjugative transposon system and phage that may aid in horizontal gene transfer.

The co-location of a number of *P. gingivalis* strains in the oral cavity provides a larger gene pool for *P. gingivalis* to draw on, and allelic exchange and recombination enables the bacterium to produce a wide range of phenotypes some of which may be more suited to the prevailing environmental conditions. This may permit *P. gingivalis* to increase in abundance by avoiding the host response or more efficiently obtaining nutrients. Rather than the presence of disease-associated strains or types, it may be the ability to achieve antigenic and/or functional variation through gene conversion that allows *P. gingivalis* to dysregulate host defenses and tip the homeostatic balance that exists within the host to trigger dysbiosis.

In conclusion, *P. gingivalis* uses specific domain rearrangements and allelic exchange through horizontal gene transfer to generate diversity in specific surface virulence factors. This diversity of specific genes and recombination means that typing schemes only provide limited useful information unless the complete diversity of *P. gingivalis* genes within an individual oral cavity or indeed periodontal pocket is captured. It is likely that the specific combination of genes in the genome of the dominant *P. gingivalis* strain may vary with the disease state and host response. Obtaining *P. gingivalis* genome sequences from strains isolated from both healthy and diseased individuals/sites may elucidate the full variety and number of strains that exist within the oral cavity.

## Author contributions

Conceived and designed the experiments: SD, HM, CS, TS, DB, and ER. Performed the experiments: HM, CS, SG, DB, PC, KC, and SC. Analyzed the data: SD, HM, CS, SG, TS, DB, PC, KC, and ER. Contributed to reagents/materials/analysis tools: SG, TS, DB, PC, KC, and ER. Wrote the paper: SD, HM, and CS. Collected the samples: HM, CS, and SC. Critically revised the manuscript: SD, HM, CS, SG, TS, DB, PC, KC, SC, and ER.

## Funding

This work was supported by the Australian Government, Department of Industry, Innovation and Science and the Australian National Health and Medical Research Council Project 1081252.

### Conflict of interest statement

The authors declare that the research was conducted in the absence of any commercial or financial relationships that could be construed as a potential conflict of interest.
