# Supplementary material for: Porphyromonas gingivalis Uses Specific Domain Rearrangements and Allelic Exchange to Generate Diversity in Surface Virulence Factors
Source: Front Microbiol. 2017 Jan 26;8:48. doi: 10.3389/fmicb.2017.00048 (PMC5266723; doi:10.3389/fmicb.2017.00048)
Supplement: Supplementary file 1 [file Table1.DOCX]

**Supplementary Table S1.** Codon positions in gingipain catalytic domains with selection signals.

| Domain | Codon position | SLAC dN-dS | SLAC p-value | FEL dN-dS | FEL p-value | FUBAR dN-dS | FUBAR Posterior Probability |
| --- | --- | --- | --- | --- | --- | --- | --- |
| RgpA_cat_ | 284 | -120.434 | 0.004 | -3205.92 | 0.029 | -5.775 | 0.91 |
| RgpA_cat_ | 292 | -43.47 | 0.13 | -2983.43 | 0.033 | -5.731 | 0.915 |
| RgpA_cat_ | 375 | -69.779 | 0.007 | -1422.35 | 0.014 | -9.886 | 0.997 |
| RgpA_cat_ | 439 | -59.655 | 0.014 | -1110.44 | 0.043 | -7.019 | 0.981 |
| RgpA_cat_ | 486 | -29.827 | 0.111 | -712.854 | 0.023 | -5.339 | 0.972 |
| RgpA_cat_ | 512 | -29.827 | 0.111 | -813.531 | 0.018 | -5.834 | 0.977 |
| RgpA_cat_ | 565 | -59.655 | 0.012 | -1021.55 | 0.023 | -6.912 | 0.984 |
| RgpB_cat_ | 281 | -41.581 | 0.111 | -113.565 | 0.053 | -3.168 | 0.961 |
| RgpB_cat_ | 286 | -113.83 | 0.015 | -1263.82 | 0 | -10.893 | 0.999 |
| RgpB_cat_ | 292 | -57.969 | 0.07 | -138.077 | 0.041 | -3.759 | 0.964 |
| RgpB_cat_ | 377 | -28.095 | 0.028 | -281.383 | 0.011 | -7.645 | 0.998 |
| RgpB_cat_ | 488 | -192.494 | 0.111 | -273.175 | 0.017 | -6.206 | 0.985 |
| RgpB_cat_ | 497 | -166.764 | 0.224 | -608.9 | 0.034 | -4.5 | 0.904 |
| RgpB_cat_ | 526 | -77.711 | 0.008 | -259.742 | 0.002 | -7.708 | 1 |
| RgpB_cat_ | 551 | -35.342 | 0.111 | -162.761 | 0.025 | -4.557 | 0.974 |
| RgpB_cat_ | 567 | -35.342 | 0.111 | -137.607 | 0.027 | -4.047 | 0.975 |
| RgpB_cat_ | 571 | -35.342 | 0.111 | -78.579 | 0.05 | -1.823 | 0.935 |
| Kgp_cat_I | 299 | -432.691 | 0.122 | -1938.67 | 0.023 | -8.338 | 0.99 |
| Kgp_cat_I | 522 | -432.691 | 0.111 | -1848.9 | 0.025 | -7.958 | 0.99 |
| Kgp_cat_I | 548 | -676.049 | 0.12 | -4196.03 | 0.025 | -6.082 | 0.926 |
| Kgp_cat_II | 555 | -157.592 | 0.12 | -5333.88 | 0.04 | -5.116 | 0.907 |

SLAC - Single likelihood ancestor counting method

FEL - Fixed effects likelihood method

FUBAR - Fast unconstrained Bayesian approximation method

dN-dS – non-synonymous and synonymous substitutions
