# Supplementary material for: Porphyromonas gingivalis Uses Specific Domain Rearrangements and Allelic Exchange to Generate Diversity in Surface Virulence Factors
Source: Front Microbiol. 2017 Jan 26;8:48. doi: 10.3389/fmicb.2017.00048 (PMC5266723; doi:10.3389/fmicb.2017.00048)
Supplement: Supplementary file 3 [file Image1.PDF]

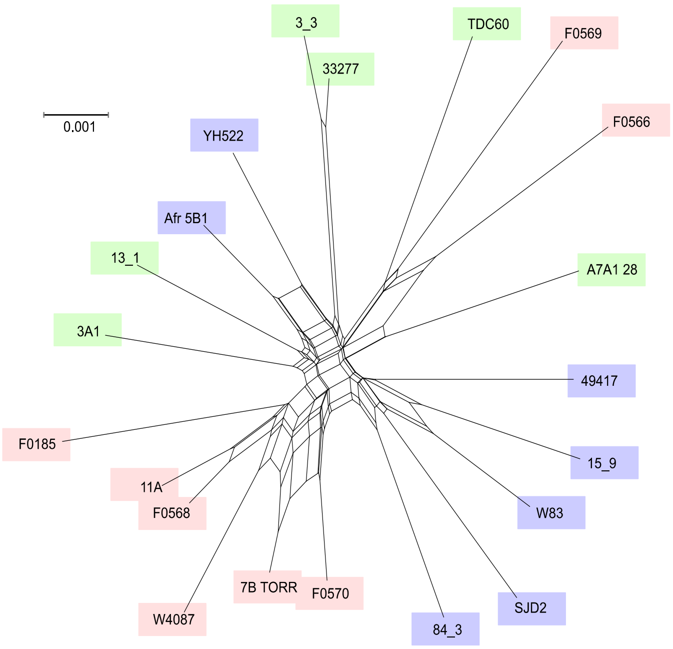

Figure S1. NeighborNet network analysis of *P. gingivalis* concatenated MLST genes. Complete sequences from six of the seven genes in the *P. gingivalis* MLST gene set were individually aligned with MAAFT. The paralogous nature of *hagB* precluded it from inclusion in this analysis. Individual alignments were than concatenated to create a single alignment of each genome. The reticulated network was generated with the NeighborNet algorithm in SplitsTree 4 using uncorrected P distances. The taxa are coloured based on the groupings defined in **Fig 1**. The distance scale in the upper left indicates the number of nucleotide substitutions per site.
