# Supplementary material for: Porphyromonas gingivalis Uses Specific Domain Rearrangements and Allelic Exchange to Generate Diversity in Surface Virulence Factors
Source: Front Microbiol. 2017 Jan 26;8:48. doi: 10.3389/fmicb.2017.00048 (PMC5266723; doi:10.3389/fmicb.2017.00048)
Supplement: Supplementary file 4 [file Image2.PDF]

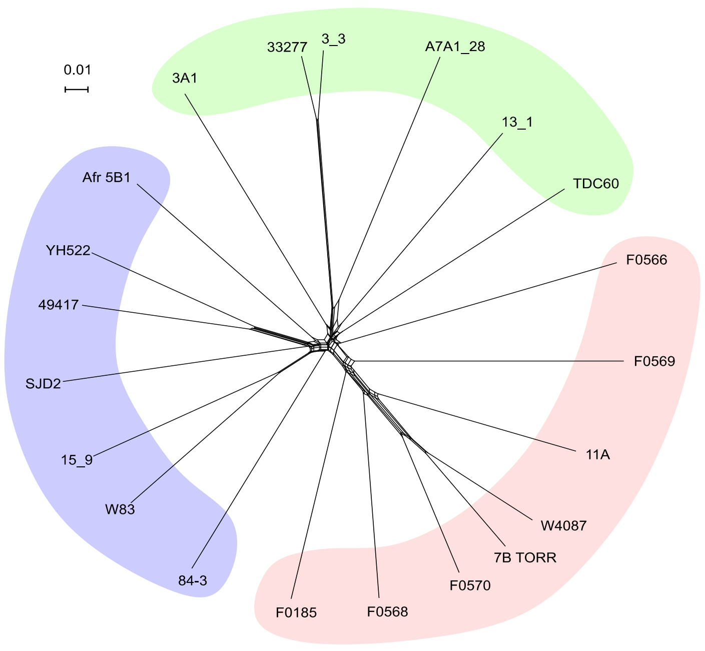

Figure S2. NeighborNet network analysis of *P. gingivalis* single nucleotide polymorphism (SNP) alignment. Draft *P. gingivalis* genomes from this research and completed genomes from the NCBI Genbank database were analysed using the Harvest suite (ver 1.0) core-genome alignment tools. Fasta DNA sequence files for each genome were aligned using Parsnp with recombination detection active (option '-x') and minimum LCB size set to 60 (option '-z 60'). An Fasta alignment of single nucleotide polymorphisms (SNPs) was output using Gingr. This alignment contains only those SNPs located in nucleotide positions present in all of the genomes examined (core genome SNPs). A NeighborNet network exhibiting only limited reticulation was generated with SplitsTree 4 using uncorrected P distances. Taxa are coloured based on the groupings defined in **Fig 1**. The distance scale in the upper left indicates the number of nucleotide substitutions per site.
