# Supplementary material for: Porphyromonas gingivalis Uses Specific Domain Rearrangements and Allelic Exchange to Generate Diversity in Surface Virulence Factors
Source: Front Microbiol. 2017 Jan 26;8:48. doi: 10.3389/fmicb.2017.00048 (PMC5266723; doi:10.3389/fmicb.2017.00048)
Supplement: Supplementary file 5 [file Image3.PDF]

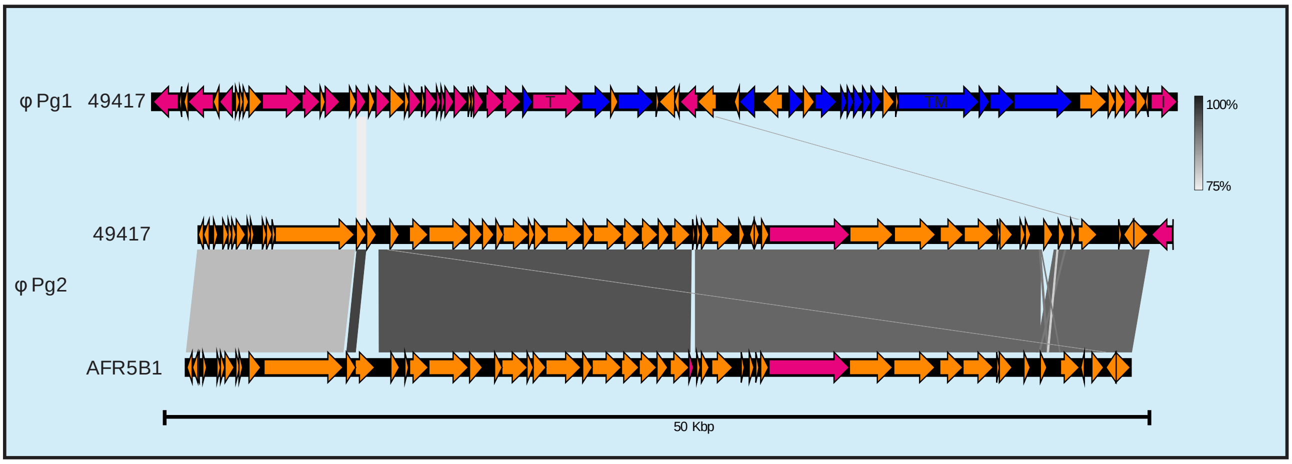

Figure S3. *P. gingivalis* prophage. ϕPg1 was identified in strain ATCC 49417 with 36 of 72 ORFs predicted to be homologs of bacteriophage genes (pink & blue). Fifteen of these ORFs were most closely related to the Riemerella bacteriophage RAP 44 (blue). A second, unrelated prophage (ϕPg2) was identified in ATCC 49417 and also found in AFR5B1.
