# Supplementary material for: Porphyromonas gingivalis Uses Specific Domain Rearrangements and Allelic Exchange to Generate Diversity in Surface Virulence Factors
Source: Front Microbiol. 2017 Jan 26;8:48. doi: 10.3389/fmicb.2017.00048 (PMC5266723; doi:10.3389/fmicb.2017.00048)
Supplement: Supplementary file 6 [file Image4.PDF]

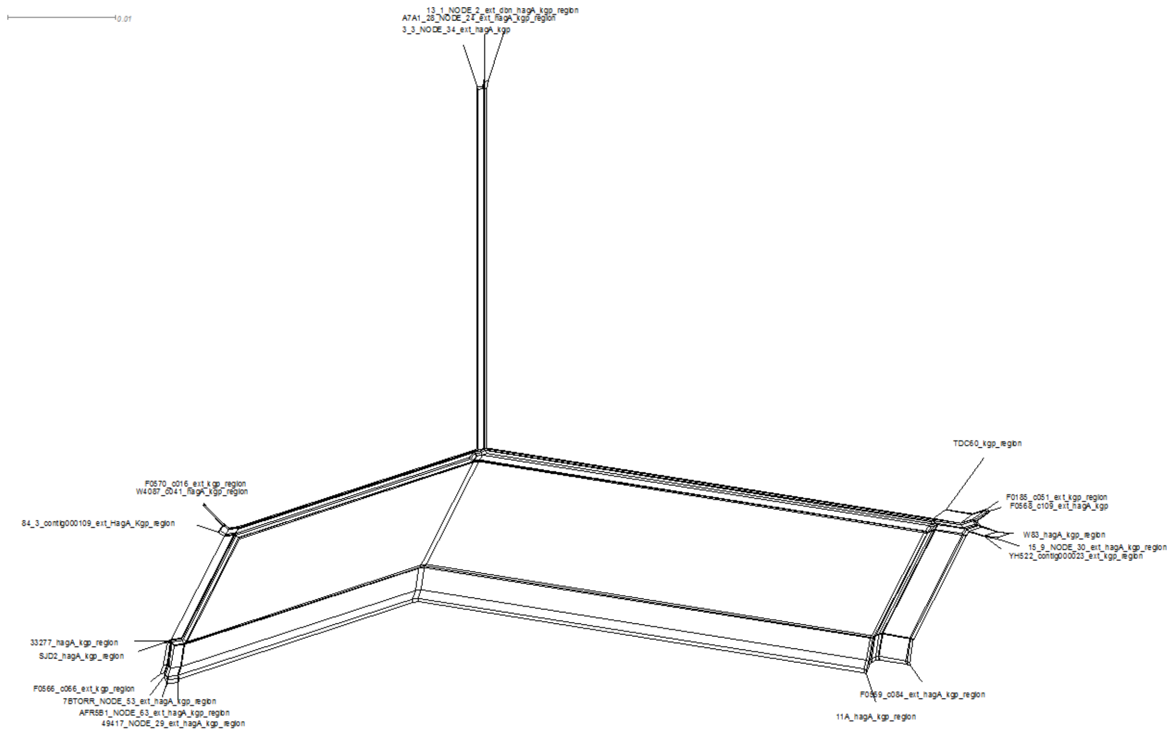

Figure S4. NeighborNet network analysis of *P. gingivalis* Kgp proteins. The *kgp* genes were extracted from the *P. gingivalis* genomes manually. The paralogous nature of the gingipains made automated assembly and annotation challenging. Therefore, *kgp* genes were assembled using the Bandage bioinformatics tool and the sequence of the 11 available strains was confirmed by PCR amplification and Sanger-sequencing. The *kgp* gene DNA sequences were converted to amino acid sequences prior to alignment with MAAFT then converted back to a DNA alignment (implemented in Geneious R8). The resulting sequence alignment was analysed with SplitsTree 4. The non-tree like appearance of the NeighborNet network (plotted with uncorrected P distances) is indicative of substantial recombination or horizontal gene transfer. The distance scale in the upper left indicates the number of nucleotide substitutions per site.
