# Supplementary figures and images for: Porphyromonas gingivalis Uses Specific Domain Rearrangements and Allelic Exchange to Generate Diversity in Surface Virulence Factors
Source: Front Microbiol. 2017 Jan 26;8:48. doi: 10.3389/fmicb.2017.00048 (PMC5266723; doi:10.3389/fmicb.2017.00048)

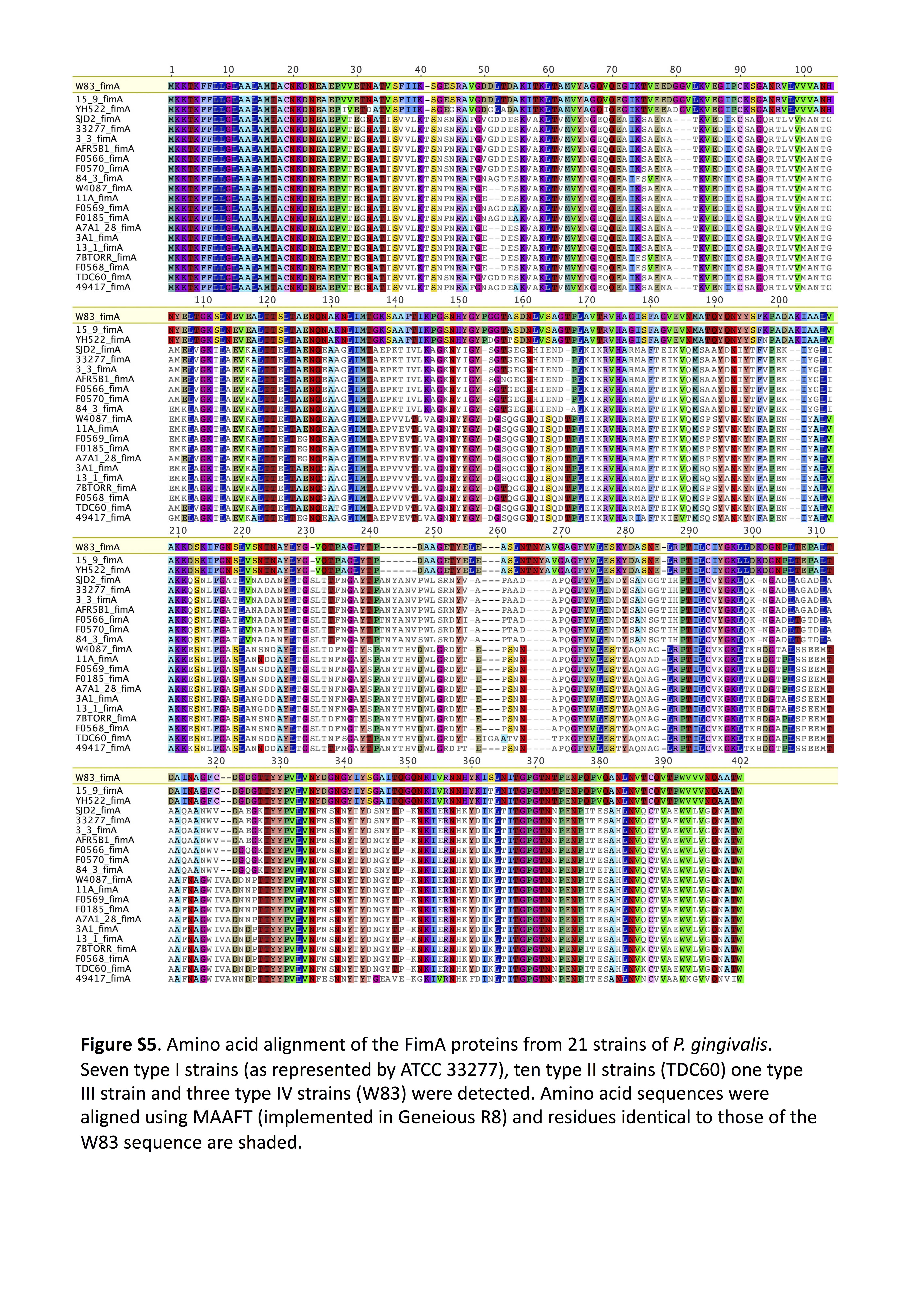

Supplement: Supplementary file 7 [file Image5.PNG]
