# Supplementary material for: Porphyromonas gingivalis Uses Specific Domain Rearrangements and Allelic Exchange to Generate Diversity in Surface Virulence Factors
Source: Front Microbiol. 2017 Jan 26;8:48. doi: 10.3389/fmicb.2017.00048 (PMC5266723; doi:10.3389/fmicb.2017.00048)
Supplement: Supplementary file 8 [file Image6.PDF]

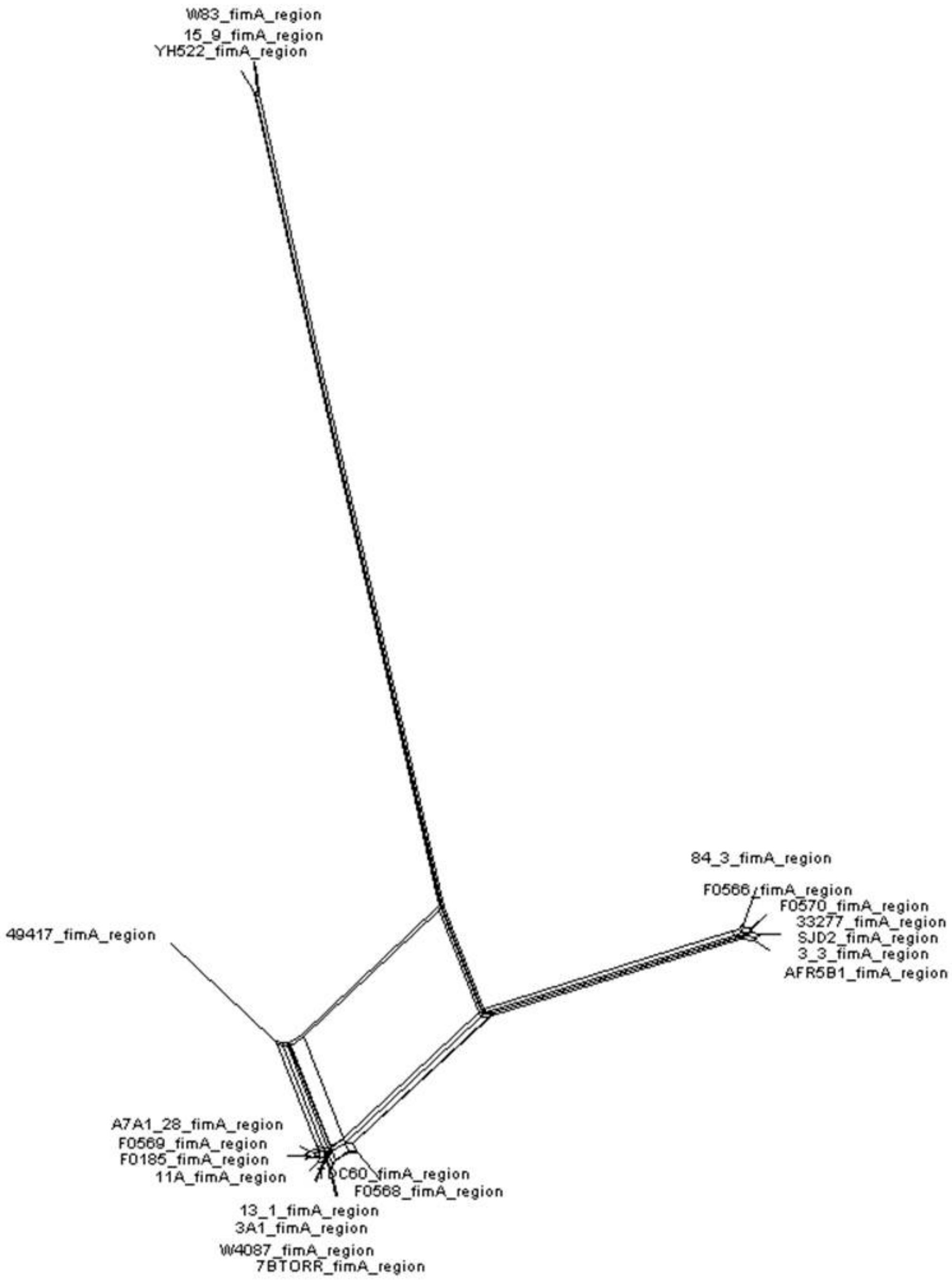

Figure S6. NeighborNet network analysis of *P. gingivalis fimA* sequences.
The *fimA* genes were extracted from the genomes manually. The single known type V sequence from strain HNA 99 (Genbank accession GI:6429668) was included for reference. Only unique sequences were included. The *fimA* gene DNA sequences were converted to amino acid sequences prior to alignment with MAAFT, then converted back to a DNA alignment (implemented in Geneious R8). The resulting sequence alignment was analysed with SplitsTree 4. A NeighborNet network was generated with uncorrected P distances. The long branch lengths and non-tree like appearance were indicative of substantial divergence between some sequences and a high likelihood of recombination or horizontal gene transfer.
