# Supplementary material for: Porphyromonas gingivalis Uses Specific Domain Rearrangements and Allelic Exchange to Generate Diversity in Surface Virulence Factors
Source: Front Microbiol. 2017 Jan 26;8:48. doi: 10.3389/fmicb.2017.00048 (PMC5266723; doi:10.3389/fmicb.2017.00048)
Supplement: Supplementary file 9 [file Image7.PDF]

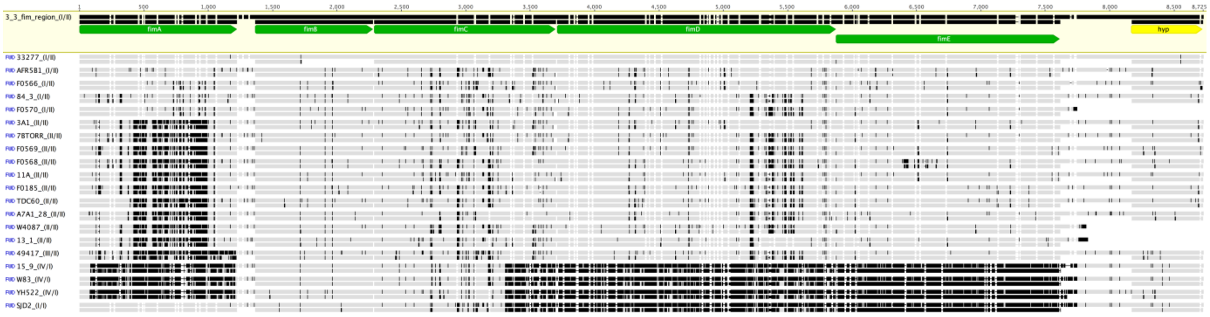

Figure S7. Alignment of the fimABCDE region from 21 strains of *P. gingivalis*. Strain 3_3 was used as the reference and any disagreements to the reference are shaded. For each strain the top line represents the DNA sequence and the bottom line represents the predicted amino acid sequence encoded by each of the fim genes. A downstream region encoding a hypothetical protein is also included. The fimA type and fimCDE types are sequentially listed in brackets after the strain name. DNA Alignment was performed using Geneious R8. Two highly divergent regions are apparent, the fimA region and a region spanning the 3' end of fimC to fimE. Signals of recombination were detected using the Phi test conducted in SplitsTree 4 (P value = 0.0). In addition, a recombination break point was identified within fimC using the DualBrothers algorithm in Geneious. Visual comparison between the fimABC of SJD2 and the sequences above clearly indicates high likelihood of recombination.
