# Supplementary material for: Porphyromonas gingivalis Uses Specific Domain Rearrangements and Allelic Exchange to Generate Diversity in Surface Virulence Factors
Source: Front Microbiol. 2017 Jan 26;8:48. doi: 10.3389/fmicb.2017.00048 (PMC5266723; doi:10.3389/fmicb.2017.00048)
Supplement: Supplementary file 10 [file Image8.PDF]

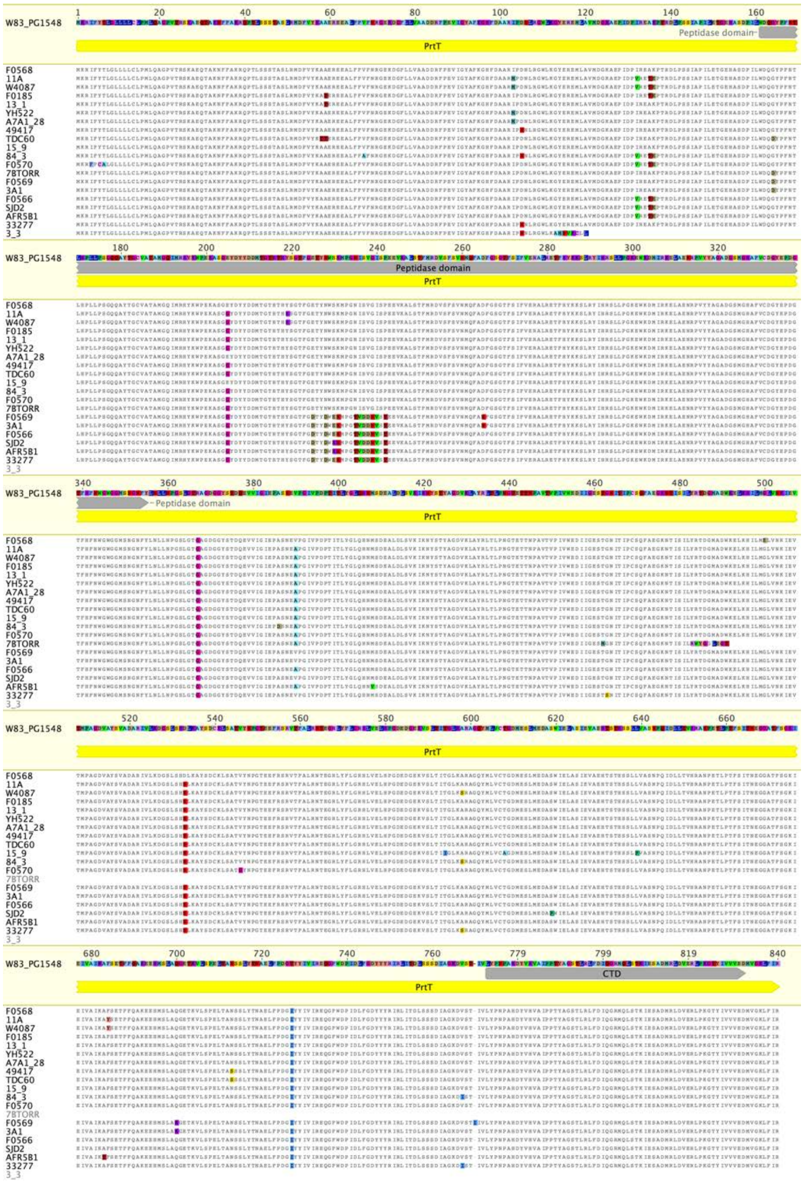

**Figure S8.** Amino acid alignment of the PrtT (PG1548) proteins from 21 strains of *P. gingivalis*. Amino acid sequences were aligned using MAAFT (implemented in Geneious R8) and residues different to that of the W83 reference are coloured. Protein domains were detected using PFAM. The PrtT protein is predicted to contain a peptidase C10 catalytic domain and a CTD domain. Two distinct types of catalytic domains are clearly present, in contrast to the high level of conservation seen throughout the rest of the protein.
