# Supplementary material for: Porphyromonas gingivalis Uses Specific Domain Rearrangements and Allelic Exchange to Generate Diversity in Surface Virulence Factors
Source: Front Microbiol. 2017 Jan 26;8:48. doi: 10.3389/fmicb.2017.00048 (PMC5266723; doi:10.3389/fmicb.2017.00048)
Supplement: Supplementary file 11 [file Image9.PDF]

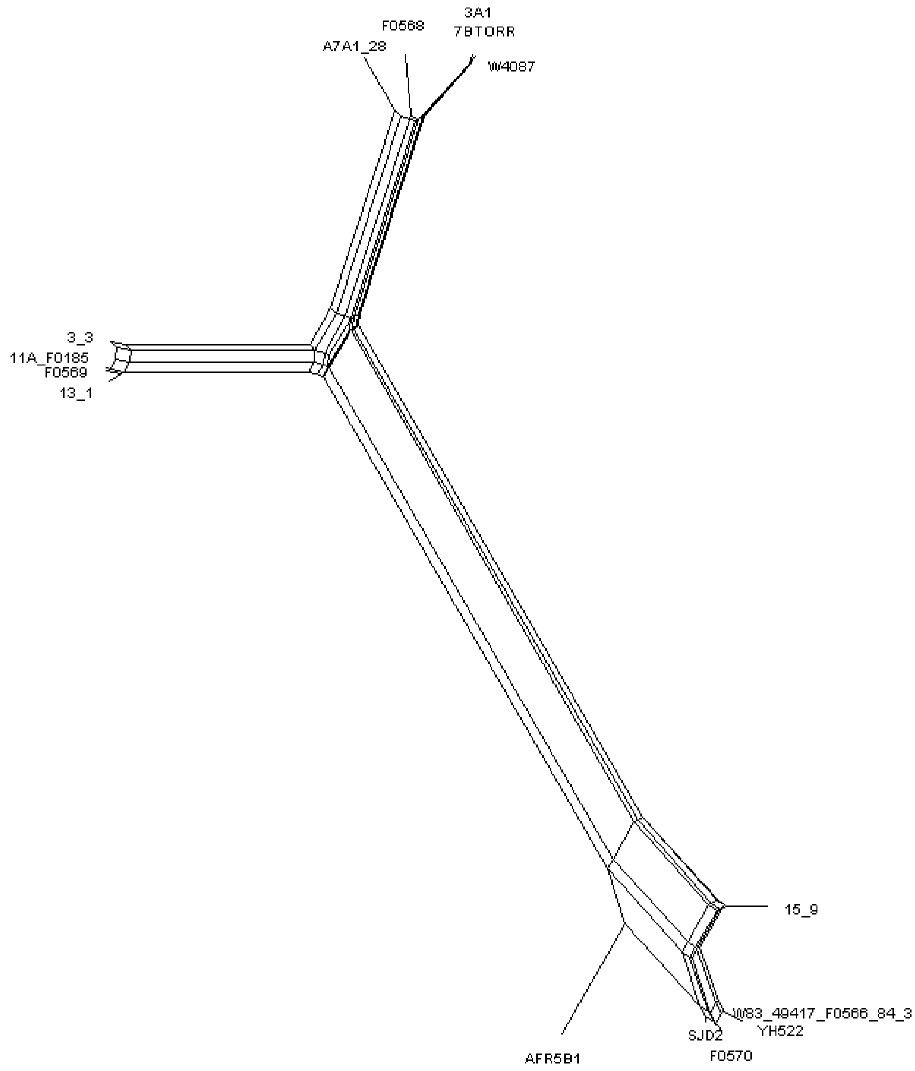

Figure S9. NeighborNet network analysis of *P. gingivalis* Tpr proteins The *tpr* genes were extracted from the *P. gingivalis* genomes manually. The *tpr* gene DNA sequences were converted to amino acid sequences prior to alignment with MAAFT then converted back to a DNA alignment (implemented in Geneious R8). The resulting DNA sequence alignment was analysed with SplitsTree 4. A NeighborNet network was generated with uncorrected P distances. The network is indicative of three clades with an uncertain phylogenetic relationship due to recombination or horizontal gene transfer.
