# Supplementary material for: Porphyromonas gingivalis Uses Specific Domain Rearrangements and Allelic Exchange to Generate Diversity in Surface Virulence Factors
Source: Front Microbiol. 2017 Jan 26;8:48. doi: 10.3389/fmicb.2017.00048 (PMC5266723; doi:10.3389/fmicb.2017.00048)
Supplement: Supplementary file 12 [file Image10.PDF]

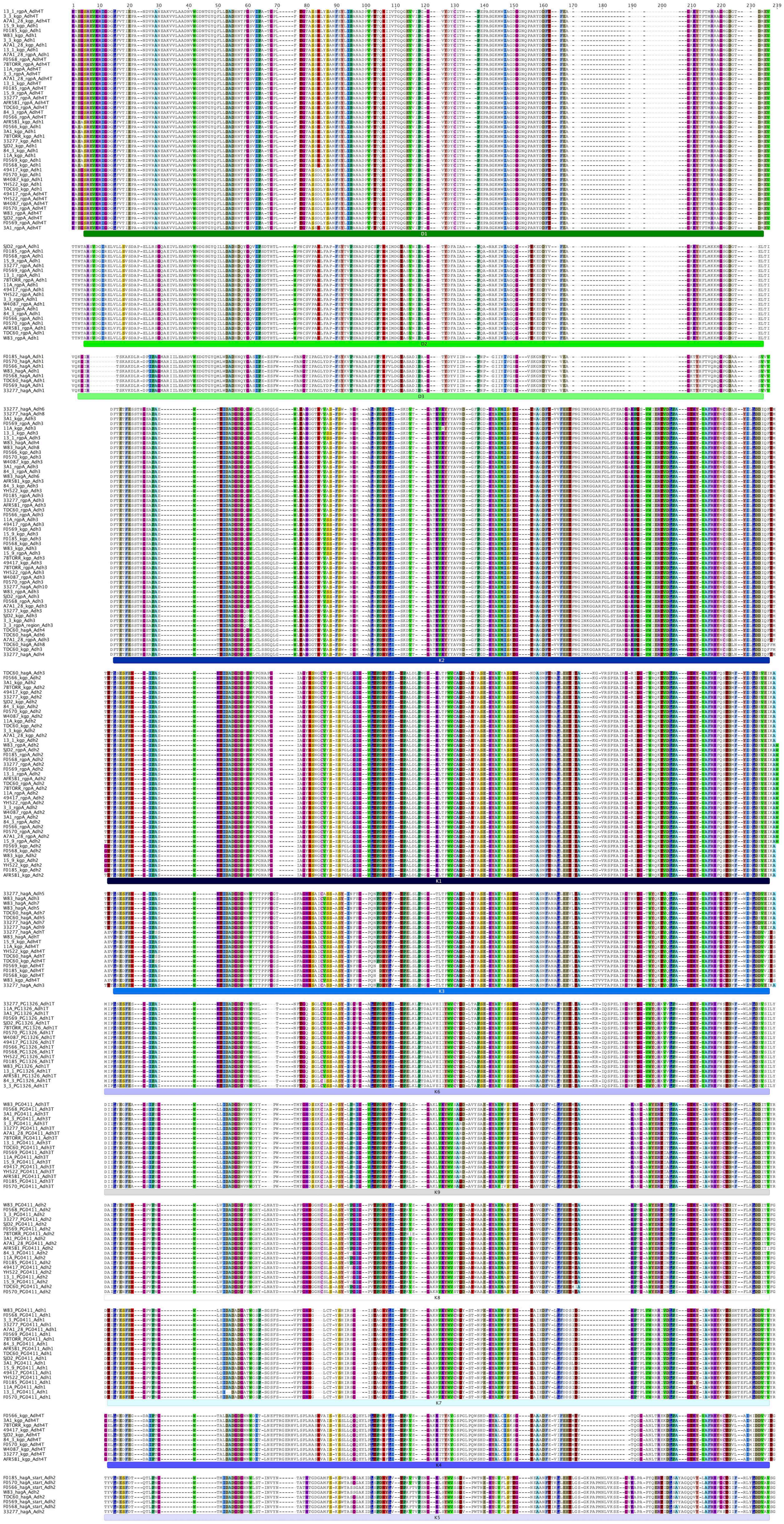

Figure S10. Amino acid alignment of the twelve distinct adhesin types found in five different proteins across twenty-one strains of *P. gingivalis*. The Kgp, RgpA, HagA, PG0411 and PG1326 proteins each contain one or more regions with identifiable CAD or DUF2436 adhesin domains. Nine distinct CAD types and three distinct DUF2436 types were found. Within each adhesin type, amino acids share 92 to 100 % identity. Between types, the most similar are K1 and K3, sharing between 67 to 74 % amino-acid identity. CAD and DUF2436 types are extremely divergent, sharing only 10 to 16 % amino-acid identity. Amino acid sequences were aligned using MAAFT (implemented in Geneious R8) and residues occurring in 25 % or more of the sequences are coloured. PFAM was used to detect CAD and DUF2436 domains and the sequence matching the hmm profile is shown by the horizontal bars.
